# Transcriptional profiling and coexpression integration for the filtering of relevant bacterial sRNA–mRNA interactions: application to *Staphylococcus aureus* biofilm

**DOI:** 10.1093/bioadv/vbag309

**Published:** 2026-10-13

**Authors:** Carolina Albuquerque Massena Ribeiro, Guadalupe del Rosario Quispe Saji, Maiana de Oliveira Cerqueira e Costa, Alice Slotfeldt Viana, Mariana Fernandes Carvalho, Agnes Marie Sá Figueiredo, Edgardo Galán-Vásquez, Jesus Eduardo Martínez-Hernández, Marisa Fabiana Nicolás

**Affiliations:** COMOD, Laboratório Nacional de Computação Científica (LNCC), Petrópolis, RJ, 256510-75, Brasil; DSC, Universidade Estadual de Campinas (UNICAMP), Campinas, SP, 13083-970, Brasil; Instituto de Microbiologia Paulo de Góes, Universidade Federal do Rio de Janeiro (UFRJ), Rio de Janeiro, RJ, 21941-902, Brasil; COMOD, Laboratório Nacional de Computação Científica (LNCC), Petrópolis, RJ, 256510-75, Brasil; Instituto de Microbiologia Paulo de Góes, Universidade Federal do Rio de Janeiro (UFRJ), Rio de Janeiro, RJ, 21941-902, Brasil; Instituto de Microbiologia Paulo de Góes, Universidade Federal do Rio de Janeiro (UFRJ), Rio de Janeiro, RJ, 21941-902, Brasil; Instituto de Microbiologia Paulo de Góes, Universidade Federal do Rio de Janeiro (UFRJ), Rio de Janeiro, RJ, 21941-902, Brasil; Instituto de Investigaciones en Matemáticas Aplicadas y en Sistemas, Universidad Nacional Autónoma de México (UNAM), Mexico, 04510, México; Núcleo de Investigación en Data Science, Facultad de Ingeniería y Negocios, Universidad de Las Américas (UDLA), Santiago, 7500975, Chile; COMOD, Laboratório Nacional de Computação Científica (LNCC), Petrópolis, RJ, 256510-75, Brasil

## Abstract

**Motivation:**

Small regulatory ribonucleic acids coordinate bacterial gene expression and contribute to biofilm development, virulence, and stress adaptation. Integrating sequence-based predictions with expression evidence can help prioritize candidate regulatory interactions for experimental investigation.

**Results:**

We characterized these regulators across six Staphylococcus aureus strains by integrating newly generated transcriptome data from Bmb9393 with public datasets from five additional strains. Sequence homology and covariance models supported candidate annotation and genomic-context characterization. Differential expression and weighted gene coexpression analysis identified conserved and strain-specific patterns, including 10 small regulatory transcripts consistently upregulated and 4 downregulated in biofilm. Combining four interaction-prediction tools with differential-expression consensus and coexpression evidence reduced 1 731 060 candidate interactions to 1290 prioritized pairs under calibrated thresholds. Comparisons across three threshold schemes showed nested target retention but substantial changes in selected regulatory partners. The 31 literature-curated interactions used for threshold calibration did not constitute independent validation, and none survived all down-stream filters. The resulting candidates provide hypotheses for experimental investigation of biofilm-associated regulation.

**Availability and implementation:**

The open-source graphical application integrates documented processed inputs and exports intermediate results for traceability. Source code, documentation, and links to study inputs and reference outputs are available at https://github.com/carolina5massena/Integrative-RNA-Seq-and-Co-expression-Analyses-Uncover-Confident-sRNA.

## 1 Introduction

Antimicrobial resistance remains a significant global public health concern, with methicillin-resistant *Staphylococcus aureus* (MRSA) representing one of the most critical threats. In Latin America, MRSA accounts for a substantial proportion of *S.aureus* infections, with regional estimates indicating that at least one-quarter of isolates are methicillin-resistant. In Brazil, national surveillance by ANVISA has consistently documented a high burden of MRSA in adult intensive care units (ICUs) and catheter-associated bloodstream infections, with recent reports showing that more than half of *S.aureus* bloodstream isolates from adult ICUs were methicillin-resistant. This burden is particularly important in ICUs, where the widespread use of invasive devices such as central venous catheters predisposes patients to biofilm-associated infections, reinforcing the urgent need for strategies that effectively address both biofilm formation and antimicrobial resistance (Seas *et al.* 2018; Boletins e relatórios—Agência Nacional de Vigilância Sanitária—Anvisa 2025).

Historically, antibacterial drug development has focused on protein-coding targets involved in essential cellular pathways, including cell-wall synthesis, DNA replication, and metabolism, and this paradigm remains the backbone of antimicrobial therapy (Nicolás *et al.* 2020). However, repeated antibiotic exposure accelerates the selection of resistant populations and shortens the clinical lifespan of antimicrobials (Belete 2019, Serral *et al.* 2021). In light of increasing bacterial resistance, therapeutic discovery should diversify the target repertoire by incorporating underexplored coding genes and ncRNA-mediated regulatory pathways. Research on ncRNAs, especially sRNAs, offers promising opportunities for studying and potentially controlling multidrug-resistant pathogens, including MRSA and members of the Enterobacterales. Bacterial sRNAs are rapid-acting, stress-responsive post-transcriptional regulators that modulate microbial metabolism and other cellular processes. Depending on target and biological context, they can inhibit translation through base pairing that occludes the Shine–Dalgarno (SD) sequence, promote translation by exposing the ribosome-binding site, enhance or prevent target-mRNA degradation, and act as molecular sponges by sequestering other sRNAs or RNA-binding proteins (Bouvier *et al.* 2008, Desnoyers *et al.* 2013, Carrier *et al.* 2018, Dutta and Srivastava 2018, Leistra *et al.* 2019, Ribeiro *et al.* 2024).

Therapeutic strategies that harness or target regulatory RNAs to reprogram bacterial gene expression represent an emerging anti-infective approach. Proof of principle for the pharmacological targeting of structured bacterial RNAs has been provided by small molecules capable of binding RNA and modulating its function. For example, ribocil, a phenotypically discovered synthetic mimic of flavin mononucleotide (FMN), selectively binds the FMN riboswitch, represses *ribB* expression, and inhibits bacterial growth, demonstrating that structured bacterial RNA elements can be pharmacologically targeted (Howe *et al.* 2015). Such strategies extend beyond conventional approaches focused exclusively on protein targets and expand the repertoire of potential mechanisms of action (Dersch *et al.* 2017, Matsui and Corey 2017, Parmeciano Di Noto *et al.* 2019, Wang *et al.* 2022). The regulatory versatility of sRNAs, combined with their ability to coordinate the regulation of multiple target genes, further highlights their potential relevance to antimicrobial resistance and bacterial adaptation (Howden *et al.* 2013, Dersch *et al.* 2017, Lalaouna *et al.* 2019, Mediati *et al.* 2021).

High-throughput RNA sequencing has transformed bacterial transcriptomics, enabling the systematic and cost-effective discovery of sRNAs. Public genome and transcriptome resources, combined with modern bioinformatics approaches, now support detailed characterization of sRNA repertoires and their potential regulatory interactions. Integrating public datasets with *in silico* approaches provides valuable insights into these molecules and their possible relevance as therapeutic targets. Nevertheless, many available datasets remain unexplored from an sRNA-centered perspective and therefore constitute a valuable reservoir for regulatory RNA discovery (Kim 2017, Batool *et al.* 2019, Liao *et al.* 2022, Zhang *et al.* 2022a).

In this context, *in silico* prediction of sRNA–mRNA interactions is essential for advancing and scaling bacterial regulatory RNA research. Methods that integrate thermodynamic modeling, comparative genomics, and machine learning can prioritize candidate sRNA–mRNA interactions at the genome scale. However, computational target predictions have intrinsic limitations: context-dependent RNA structures, RNA-binding proteins, transcript abundance, and RNA modifications may contribute to both false-positive and false-negative predictions. Consequently, prioritization frameworks should integrate multiple independent layers of evidence and produce experimentally tractable candidates for subsequent *in vitro* and *in vivo* validation (Li *et al.* 2012, Georg *et al.* 2020).

Here, we address these challenges through a reproducible integrative framework that combines sequence-based sRNA–mRNA interaction prediction with differential-expression evidence, coexpression network topology, and cross-strain consistency. We applied this framework to newly generated RNA-seq data from the Brazilian ST239 strain Bmb9393 of *S. aureus* and reanalyzed public datasets from five US-lineage strains. We mapped candidate sRNA loci, quantified their expression under planktonic and biofilm-promoting conditions, and integrated differential gene expression (DEG) with weighted gene coexpression network analysis (WGCNA) to identify regulatory modules. Candidate interactions predicted by IntaRNA, RNAplex, TargetRNA3, and sRNARFTarget were progressively prioritized using calibrated tool-specific thresholds, cross-strain differential-expression consensus, WGCNA same-module and network-edge requirements, and a final target-level selection retaining the highest-weight sRNA for each target. This framework reduced 1 731 060 initial candidate interactions to a final set of 1290 prioritized sRNA–mRNA pairs under the calibrated scheme. The resulting dataset highlights regulatory candidates associated with biofilm formation, stress responses, antimicrobial resistance, and metabolism, providing experimentally tractable hypotheses across diverse genetic backgrounds.

## 2 Methods

To characterize small RNAs (sRNAs) and prioritize candidate sRNA–mRNA interactions in *S. aureus*, we followed four main steps: (i) we identified and curated sRNAs in strain Bmb9393 and in additional strains with incomplete ncRNA annotations; (ii) we analyzed RNA-seq data from planktonic and biofilm conditions using differential expression analysis and weighted gene coexpression network analysis (WGCNA); (iii) we predicted sRNA–mRNA interactions using the Bmb9393 reference genome; and (iv) we integrated the resulting evidence through a customized, in-house graphical user interface (GUI) that implements a multicriterion filtering workflow to prioritize candidate sRNA–mRNA pairs for downstream experimental validation. Figure 1 summarizes the workflow; full details are provided below.

**Figure 1:**
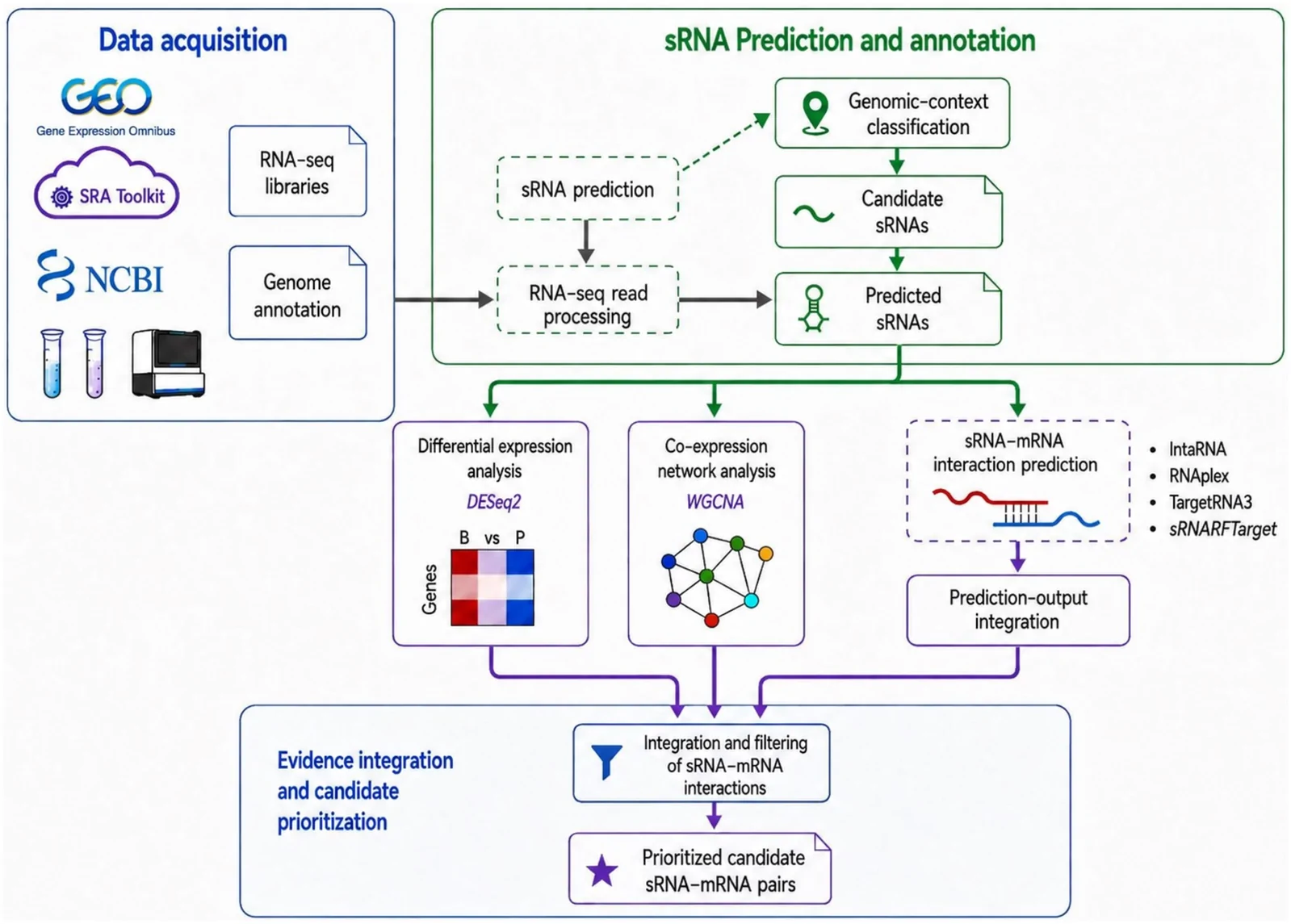
Workflow for characterizing sRNAs and prioritizing candidate sRNA–mRNA interactions in *S.aureus*. (i) Data acquisition: RNA-seq datasets generated in-house or retrieved from GEO/SRA, together with the corresponding genome sequences and annotations used in this study (Table 18, available as supplementary data at *Bioinformatics Advances* online). (ii) sRNA prediction and annotation: strand-aware identification of candidate sRNAs and their classification according to genomic context as intergenic, intragenic, antisense, or UTR-associated sRNAs. (iii) RNA-seq processing and expression analysis: processing of raw reads to obtain gene- and sRNA-level counts, followed by differential expression analysis between biofilm and planktonic conditions. (iv) Target prediction: putative sRNA–mRNA interactions were independently predicted using IntaRNA, RNAplex, TargetRNA3, and sRNARFTarget, followed by integration of their outputs. (v) Coexpression network analysis: WGCNA modules and network export using a Cytoscape edge-weight threshold of 0.1. (vi) Evidence integration and candidate prioritization: the GUI integrates sequence-based predictions, differential-expression results, and coexpression evidence. Candidate pairs must satisfy the selected filtering criteria, including same-module assignment and presence of their connecting edge in the exported network. No additional downstream minimum edge-weight cutoff was applied in this study. Final target-level prioritization retains the highest-weight sRNA partner for each target mRNA. Dashed-outline boxes indicate procedures performed outside the GUI: initial sRNA prediction, raw-read processing, and execution of the external target-prediction programs. The GUI imports processed inputs for subsequent analyses, integration, filtering, and prioritization. B, biofilm; P, planktonic.

### 2.1 Identification of sRNAs

To identify sRNA loci in this study, two distinct approaches were employed. The first method relied on sequence homology detection using the nonredundant sequences available at the NR2 (https://nr2.ncrnadatabases.org) and Staphylococcal Regulatory RNAs Database (SRD; Sassi *et al.* 2015) databases. The second approach involved predicting and annotating sRNAs using the StructRNAFinder pipeline, based on secondary-structure covariance models derived from known RNA families (Arias-Carrasco *et al.* 2018). Results from the two approaches (sequence homology and covariance model predictions) were combined for consolidation. For consistency, sRNAs were named according to the most common name entry in the SRD (https://srd.genouest.org).

Subsequently, we classified each predicted sRNA by genomic context relative to annotated genes in the study genomes: Bmb9393, N315, MRSA252, LAC (USA300/FPR3757), and MW2. Because a well-annotated reference for NRS385 (USA500) is unavailable, we used the LAC (USA300/FPR3757) genome as a proxy for USA500, consistent with prior studies (Li *et al.* 2009, Glaser *et al.* 2016, Tomlinson *et al.* 2021). The sRNAs were categorized into five genomic locations: (i) antisense: located on the opposite strand of a CDS, (ii) intragenic: situated within a CDS on the same strand, (iii) 5′ UTR: located upstream of a gene, (iv) 3′ UTR: located downstream of a gene, and (v) intergenic: situated between genes outside the defined UTR regions.

### 2.2 Bacterial growth under biofilm and planktonic conditions

For biofilm formation, the Bmb9393 strain was streaked on a TSA plate and incubated for 24 h at 37°C. Approximately three colonies were inoculated into 2 ml of TSB supplemented with 1% (w/v) glucose (TSB-Glu) and incubated for 24 h at 37°C with shaking at 250 rpm. The resulting cultures were diluted 1:100 in TSB-Glu, and 200 µl aliquots were dispensed into 48 wells of sterile 96-well plates (Nunclon untreated; Nunc A/S, Roskilde, Denmark), followed by a 24-h incubation at 37°C. After removal of supernatants, biofilms were washed with sterile water to remove nonadherent cells. Biofilms were then recovered by scraping with sterile toothpicks into 200 µl of TSB-Glu and immediately treated with an equal volume of cold acetone–ethanol solution (1:1, v/v). Samples were stored at −80°C until RNA preparation. For planktonic cell preparation, 18-h cultures of Bmb9393 were diluted 1:100 in TSB-Glu and incubated for 24 h at 37°C with shaking at 250 rpm. Cells were harvested, mixed with an equal volume of cold acetone–ethanol solution (1:1, v/v), and stored at −80°C until RNA preparation. A qRT-PCR experiment was performed in biological triplicate, with three technical replicates per condition. The RNA-seq experiment was performed in biological quadruplicate (Ferreira *et al.* 2012, Beltrame *et al.* 2015, Lade *et al.* 2019).

### 2.3 RNA extraction

Frozen bacterial cell suspensions in acetone–ethanol solution were thawed on ice and centrifuged at 8700× g at 4°C. The pellet was washed twice with TES buffer (20 mM Tris-HCl, pH 7.6, containing 10 mM EDTA, pH 8.0, 50 mM NaCl, and 20% wt/vol sucrose), using half the original volume of the cell suspension, followed by centrifugation. Bacterial cells were adjusted to an optical density of 0.8 at 600 nm (∼10^8^–10^9^ CFU/mL) using a SpectroMax Plus 384 spectrophotometer (Molecular Devices, Silicon Valley, CA, USA). The bacterial pellet was then resuspended in 10 µl of a 10 U/µl lysostaphin solution and 90 µl TES buffer. The mixture was incubated on ice for 30 min, followed by 5 min at 37°C (Beltrame *et al.* 2015). After bacterial lysis, RNA extraction was performed using the RNeasy Mini Kit protocol (Qiagen, Hilden, DE, Germany). The quantity of extracted RNA was measured with a NanoDrop spectrophotometer (Thermo Fisher Scientific, Waltham, MA, USA). A standard PCR amplification was performed with rrs primers (Table 19, available as supplementary data at *Bioinformatics Advances* online) to ensure the absence of DNA in the RNA preparations. The integrity of total RNA was initially assessed by visualizing the 23S/16S rRNA banding pattern using 1.2% agarose gel electrophoresis in 1× TAE buffer (20 mM Tris-acetate, 0.5 mM EDTA, pH 8.0) run at 110 V for 50 min. The gel was stained with ethidium bromide and visualized using a gel documentation system (Gel-Doc EZ Imager, Bio-Rad, Hercules, CA, USA).

### 2.4 RNA-Seq analysis of paired-end libraries

The Qiagen Stranded RNA Library Kit, which preserves strand-specific information and enables simultaneous capture of both sRNA and mRNA transcripts, was used to construct the libraries of the *S. aureus* Bmb9393 strain. Next, sequencing was performed on the Illumina NextSeq 500/550 using the NextSeq 500/550 v2 kit (75 cycles) at the UGCDFA of the Laboratório Nacional de Computação Científica (https://www.labinfo.lncc.br/ugc), Brazil. In addition, we analyzed the paired-end data from a large-scale RNA expression profiling experimentation (Tomlinson *et al.* 2021), which conducted a comprehensive transcriptomic analysis of five MRSA strains: N315 (USA100), MRSA252 (USA200), LAC or USA300 FPR3757 (USA300), MW2 (USA400), and NRS385 (USA500) (Tomlinson *et al.* 2021; see dataset description in Table 18, available as supplementary data at *Bioinformatics Advances* online). To perform a comparative analysis with the Bmb9393 strain, we retrieved data from the NCBI Gene Expression Omnibus (GEO; Edgar 2002) for the 24-h growth time point in both conditions using the SRA Toolkit (https://github.com/ncbi/sra-tools). The quality of the raw reads was first evaluated with FastQC, followed by adapter trimming and quality filtering with FastP (Chen *et al.* 2018). The quality of the processed reads was then reassessed using FastQC to ensure data integrity (https://github.com/s-andrews/FastQC). High-quality reads were aligned to the reference genome provided in FASTA format and annotated with a GTF file using Rsubread (Liao *et al.* 2019). The alignment files were subsequently processed with SAMtools (Danecek *et al.* 2021), including conversion to BAM format, sorting, and indexing. Finally, gene-level expression was quantified using FeatureCounts (Liao *et al.* 2014), which incorporated genome annotations to generate a read count matrix for downstream analyses. The raw count matrix generated during preprocessing was used for differential expression analysis between biofilm and planktonic conditions using the nf-core/differential-abundance pipeline (Ewels *et al.* 2020).

### 2.5 Real-Time qRT-PCR

Transcripts were quantified using the real-time qRT-PCR method and the Power SYBR Green RNA-to-CT™ 1-Step kit (Applied Biosystems, Foster City, CA, USA), following the manufacturer’s instructions, on a StepOne Real-Time PCR System (Applied Biosystems, São Paulo, SP, Brazil). The rrs transcript encoding 16S rRNA served as an endogenous control. Results were normalized, with the calibration sample assigned a value of 1.0. Primers were designed using Beacon Designer for SYBR® Green (https://www.premierbiosoft.com/molecular_beacons/) and are listed in Table 19, available as supplementary data at *Bioinformatics Advances* online. Gene expression data ($2^{-\Delta \Delta C_{t}}$) were log_2_-transformed before statistical analysis, with the reference group (1) set to 0. Normality was assessed using the Shapiro–Wilk test, followed by either a one-sample *t*-test or a Wilcoxon signed-rank test against 0, as appropriate. Results are presented as mean ± SD of log_2_-transformed values.

### 2.6 Construction and analysis of the coexpression network using WGCNA

The count matrix generated during preprocessing was normalized to ensure accurate and reliable downstream analyses. The TMM (Trimmed Mean of *M* values) normalization method (Robinson and Oshlack 2010) was applied to correct compositional differences across samples in the first step. After adjusting library sizes and reducing technical biases with TMM, RPKM (Reads Per Kilobase per Million mapped reads) normalization was performed. This additional step accounted for gene length and sequencing depth, providing a more balanced expression profile (Zhao *et al.* 2020). The entire normalization approach was executed in R using the edgeR (4.4.1) package (Chen *et al.* 2025). To minimize batch effects from different experiments, an additional analysis was executed using the limma package (v3.62.1) (https://bioconductor.org/packages/limma) in R. Genes with zero counts in >75% of the samples were excluded to avoid biases from low-expression genes.

The resulting expression matrix was used as input for WGCNA (Langfelder and Horvath 2008). To mitigate potential biases introduced by excessively high expression values, a log2 transformation was applied, resulting in a more balanced distribution (Zhu *et al.* 2022). The coexpression network was constructed by calculating an adjacency matrix that quantifies the connection strength between genes based on expression similarity. A crucial parameter in this process was the selection of a soft threshold power of 4, which ensured the network’s scale-free topology, a key property for maintaining a power-law distribution of gene connection degrees. The adjacency matrix was then converted into a topological overlap matrix (TOM), which captures direct and indirect connections among genes and provides a consistent view of coexpression networks (Langfelder and Horvath 2008, Soberanes-Gutiérrez *et al.* 2022).

Hierarchical clustering was performed on the TOM to group genes into modules based on their topological overlap. Distinct modules were identified using the dynamic tree-cut method applied to the resulting dendrogram. This method precisely identified coexpressed gene modules, representing groups of genes with highly correlated expression patterns. Module eigengenes were calculated to summarize the expression profiles of genes within each module. These eigengenes facilitated correlations with external traits or phenotypes of interest, such as biofilm and planktonic conditions (Langfelder and Horvath 2008, Soberanes-Gutiérrez *et al.* 2022).

The biological functions of significant modules were inferred using Gene Ontology (GO) enrichment analysis of the genes within each module. This analysis identified associated biological processes, molecular functions, and cellular components, offering detailed insights into the roles of genes within each module (Soberanes-Gutiérrez *et al.* 2022). For GO enrichment, the N315 strain was selected due to its superior annotation in UniProt, with 2583 entries in UniProtKB (https://www.uniprot.org/taxonomy/158879). Using the ClueGO app (Bindea *et al.* 2009) in Cytoscape (Shannon *et al.* 2003), modules were enriched individually for GO terms. Only terms with a *P*-value below .05 were retained. The resulting spreadsheets for each module were consolidated into a single document and used for visualization.

To identify the top hub genes within each module, the chooseTopHubInEachModule function from the WGCNA package was employed (Langfelder and Horvath 2008). This function determines the most connected gene in each module based on intramodular connectivity, which reflects a gene’s correlation with other genes in the same module. Modules labeled as “grey,” representing unassigned or noise-associated genes, were excluded from the analysis to focus on biologically relevant groups. After identifying the top hub genes, each was individually searched in the UniProt database to retrieve detailed functional annotations. In addition, GO terms were collected using QuickGO (Binns *et al.* 2009).

### 2.7 Computational prediction, evidence integration, and prioritization of sRNA–mRNA pairs

Putative sRNA–mRNA interactions were independently predicted using four complementary programs: IntaRNA (v2.0; Mann *et al.* 2017), TargetRNA3 (v3.0; Tjaden 2023), RNAplex (v2.7; Tafer and Hofacker 2008), and sRNARFTarget (v1.0; Naskulwar and Peña-Castillo 2022).

Because these tools rely on distinct algorithmic assumptions and report different types of scores, their outputs were treated as complementary rather than directly comparable (Table 1). Moreover, none of the four programs incorporates transcript abundance or condition-specific expression. Sequence-based predictions were, therefore, subsequently integrated with differential-expression and coexpression evidence.

**Table 1:** Overview and comparison of sRNA–mRNA target prediction tools used in this study.

Tool	Algorithmic principle	Required inputs	Main strengths	Key limitations
**IntaRNA (v2.0)**	Thermodynamic hybridization model incorporating accessibility of interaction sites	sRNA sequences, mRNA sequences (genome-wide)	High accuracy for energetically favorable RNA–RNA interactions; accounts for local RNA structure and binding energy	Does not consider expression context; may generate many energetically plausible but biologically irrelevant interactions
**TargetRNA3 (v3.0)**	Machine learning approach integrating seeds, conservation, and structural accessibility	sRNA sequences, annotated genome	Effective at identifying interactions near translation initiation regions; supports genome-wide scans	Biased toward 5′ UTR interactions; limited for CDS-internal or noncanonical binding sites; high false-positive rate
**RNAplex (v2.7)**	Fast energy minimization for RNA–RNA duplex formation	sRNA sequences, mRNA sequences	Computationally efficient; suitable for large-scale predictions	Simplified energy model; higher false-positive rate when used alone
**sRNARFTarget (v1.0)**	Machine-learning classifier based on sequence-derived features	sRNA sequences, mRNA sequences	Captures nonobvious interaction patterns beyond simple complementarity	Dependent on training data; predictions lack mechanistic interpretability

The query set comprised sRNAs detected in Bmb9393 with a normalized abundance >10, according to the expression-processing procedure described previously. The Bmb9393 reference annotation, including CDSs and the curated sRNA loci identified in this study, was used to generate the corresponding mRNA target and sRNA query sequences. All prediction programs were executed using their default parameters, except for IntaRNA, for which the IntaRNAsTar personality was selected. This configuration uses parameters optimized for large-scale, genome-wide sRNA target prediction (Tieng *et al.* 2023).

The outputs generated by the four programs were imported into the in-house graphical workflow, which standardized output fields and identifiers, resolved redundant records, and merged predictions into a unified sRNA–mRNA candidate table. For the analyses reported in this study, a candidate pair was required to be supported by all four prediction tools and to satisfy the corresponding score threshold for each program; thus, a four-tool AND rule was applied throughout.

To evaluate the sensitivity of candidate prioritization to the sequence-based score thresholds, the complete workflow was run under three schemes: calibrated tool-specific, restrictive common-threshold, and highly restrictive common-threshold (Table 2). The calibrated tool-specific scheme was defined using a curated set of 31 experimentally supported sRNA–mRNA interactions reported in the literature (Table 20, available as supplementary data at *Bioinformatics Advances* online). These literature-curated interactions were used to define the tool-specific sequence-based score thresholds. For each program, the threshold corresponded to the least favorable score required to retain the complete positive-control set: sRNARFTarget probability ≥0.45, IntaRNA *P*-value ≤.41, RNAplex *P*-value ≤.76, and TargetRNA3 *P*-value ≤.89. Because these interactions were used for threshold calibration and originated from different experimental contexts, they did not constitute an independent validation set or a condition-specific gold standard for biofilm regulation.

**Table 2:** Filter parameters and stepwise attrition of sRNA–mRNA interactions under the three threshold schemes.^a^

Pipeline parameter/filtering stage	Calibrated tool-specific	Restrictive common-threshold	Highly restrictive common-threshold
**WGCNA Cytoscape export threshold**	0.1	0.1	0.1
**Minimum sRNARFTarget probability**	0.45	0.50	0.60
**Maximum IntaRNA P-value**	0.41	0.30	0.10
**Maximum RNAplex P-value**	0.76	0.30	0.10
**Maximum TargetRNA3 P-value**	0.89	0.30	0.10
**Fail minimum sRNARFTarget probability**	242 860	799 179	1 720 681
**Fail maximum IntaRNA P-value**	178 020	283 091	1 396 660
**Fail maximum RNAplex P-value**	483 329	1 214 850	1 558 231
**Fail maximum TargetRNA3 P-value**	207 207	1 224 186	1 596 720
**Total input pairs**	1 731 060	1 731 060	1 731 060
**Sequence-based score consensus (4/4 tools)**	949 716	115 764	148
**DEG: after padj ≤ 0.05**	739 367	92 230	105
**DEG: after baseMean ≥ 10**	619 034	84 320	100
**DEG: after presence consensus**	149 545	23 265	28
**WGCNA: same module**	9084	1462	1
**WGCNA: requires a network edge**	8618	1373	1
**Best pair per target**	1290	567	1
**Final prioritized interactions**	1290	567	1

a All workflows use the same input dataset (1 731 060 pairs), consensus across all four prediction tools (AND rule), and downstream criteria. DEG filtering applies to all six datasets: sRNA Padj ≤ .05, baseMean ≥ 10, no absolute log2FC threshold, and presence in at least five of six strain-specific comparisons. Both partners must be in the same WGCNA module, and their edge must be present in the network exported at threshold 0.1. The additional downstream minimum edge-weight filter is off. Selection uses best_per_target (one highest-weight sRNA partner per target); the genomic-context filter is off. The four failure counts are independent and may overlap; they must not be summed. Retention counts are sequential. Final sets are provided in Tables 15–17, available as supplementary data at Bioinformatics Advances online.

Two additional schemes were applied as threshold-sensitivity analyses. In the restrictive common-threshold scheme, candidates were required to have an sRNARFTarget probability ≥.50 and *P*-values ≤.30 for IntaRNA, RNAplex, and TargetRNA3. In the highly restrictive common-threshold scheme, the corresponding cutoffs were an sRNARFTarget probability ≥.60 and *P*-values ≤.10 for each of the other three programs. The four-tool consensus requirement and all subsequent expression, coexpression, and prioritization criteria were kept identical across the three schemes, thereby isolating the effect of sequence-based threshold stringency.

Following sequence-based filtering, candidate pairs were integrated with the strain-specific differential-expression results. A pair was retained when its sRNA component had an adjusted *P*-value (*P*_adj_) ≤.05 and a DESeq2 mean normalized count (baseMean) ≥10 and was present among the DE sRNAs in at least five of the six strain-specific biofilm-versus-planktonic comparisons. This cross-strain consensus criterion allowed one strain not to meet the differential-expression requirements. No absolute log2 fold-change threshold was applied.

Coexpression support was subsequently incorporated using the Bmb9393 WGCNA network, exported with a Cytoscape edge-weight threshold of 0.1 for all three threshold schemes. Candidate pairs were retained only when the sRNA and its predicted mRNA target were assigned to the same coexpression module and their connecting edge was present in the exported edge file. No additional minimum edge-weight cutoff was applied during downstream filtering. When more than one sRNA remained associated with the same mRNA target, the pair with the highest sRNA–mRNA coexpression edge weight was retained, yielding a single selected sRNA partner per target. Finally, the genomic context of each retained sRNA—intergenic, intragenic, antisense, or UTR-associated—was appended to the output to support biological interpretation and downstream experimental prioritization. Genomic context was not used as an exclusion criterion in the analyses reported here.

Pairwise overlap among the final interaction sets generated under the calibrated tool-specific, restrictive common-threshold, and highly restrictive common-threshold schemes was calculated using the Jaccard index J(A, B) = |A ∩ B|/|A ∪ B|. Because the final target-level prioritization step retained the highest-weight sRNA partner for each target mRNA (best_per_target), the Jaccard index was calculated separately for three set definitions: exact sRNA–mRNA interaction pairs, target mRNAs, and sRNAs. Exact interaction-pair overlap required identical sRNA and mRNA partners, whereas target-mRNA and sRNA overlaps were calculated independently using the unique target and sRNA identifiers, respectively, represented in each final interaction set.

### 2.8 Graphical application for reproducible sRNA–mRNA prioritization

To enable reproducible analyses without requiring programming expertise, the integrative workflow was implemented as an open-source, cross-platform graphical application developed in Python, with an R backend for differential-expression analysis using DESeq2 and weighted gene coexpression network analysis using WGCNA. The application comprises five linked modules: (i) sRNA genomic-context classification; (ii) weighted gene coexpression network analysis; (iii) differential-expression analysis; (iv) integration of predictions generated by IntaRNA, TargetRNA3, RNAplex, and sRNARFTarget; and (v) sequential filtering and prioritization of candidate sRNA–mRNA interactions. The application imports processed count matrices and prediction outputs; upstream raw-read processing and execution of the four external target-prediction programs are performed separately. The commands used for these external steps are provided in the AUXILIARY_COMMANDS.sh file in the repository. Progress information, diagnostic messages, and intermediate outputs are displayed within the interface throughout the analysis.

The application requires Python ≥3.10 and R ≥4.1. Python dependencies are specified in requirements.txt using minimum versions (PySide6 ≥6.5, pandas ≥1.5, numpy ≥1.23, and openpyxl ≥3.0) and installed in a local virtual environment by the setup scripts. The setup scripts install R dependencies through install_packages.R using CRAN and Bioconductor, without locking package versions. The repository documents the software versions used for the reported analyses; these provide a record of the analysis environment and are not enforced by the installation scripts.

The modules can be configured for application to different organisms, datasets, and analytical stringencies. The coexpression module allows users to define the correlation measure, soft-thresholding power, network type, minimum module size, and module-merging threshold. The differential-expression module accepts a user-defined experimental design and can perform independent comparisons across sample groups, producing a separate output table for each strain or comparison. The prediction-integration module allows program-specific score thresholds to be specified and the required level of agreement among prediction tools to be defined.

The filtering module takes a combined sRNA–mRNA prediction table, differential-expression tables, and WGCNA node and edge files as input. It allows individual criteria to be enabled, disabled, and parameterized while preserving their biologically motivated order. Configurable parameters include the sequence-based score thresholds and number of criteria or tools required; adjusted-*P*-value and minimum-expression cutoffs; optional fold-change constraints; the type of cross-strain consensus and the number of strain-specific comparisons required; WGCNA module and edge requirements; minimum edge-weight and best-partner-selection rules; and the genomic-context categories to be retained. Although the application supports alternative configurations, the study-specific settings described above and summarized in Table 2 were used to generate the results reported here.

For reproducibility and traceability, the application exports the candidate set after each filtering stage, allowing the effect of individual criteria to be quantified and each final candidate to be traced back through the workflow. A reduced example dataset is distributed with the software. The complete study-specific inputs and reference outputs are available at the locations specified in the Data Availability section.

## 3 Results and discussion

### 3.1 Identification of sRNAs

The first major outcome of this study was the genome-wide annotation of noncoding sRNAs in the *S. aureus* Bmb9393 genome, achieved here for the first time. In addition, an in-house Python script was developed to classify systematically predicted sRNA loci according to their genomic context. We applied the script to five additional strains, providing a consistent annotation framework for subsequent analyses of sRNA expression and target mRNA prediction (see Tables 1–5, available as supplementary data at *Bioinformatics Advances* online). Since a well-annotated reference genome for NRS385 USA500 is not yet available, we used the LAC USA300 reference genome, as it represents a closely related available genome, in agreement with previous reports (Li *et al.* 2009, Glaser *et al.* 2016, Tomlinson *et al.* 2021). This standardized annotation enabled subsequent comparative analyses of expression patterns, orthology relationships, and candidate sRNA–mRNA interactions across all strains included in this study. Table 3 summarizes the number of sRNAs identified in each category.

**Table 3:** Distribution of predicted sRNAs according to genomic context across studied *Staphylococcus aureus* strains.

Location	Bmb9393	N315	MRSA252	LAC	MW2
**Antisense**	197	197	206	195	214
**Intragenic**	58	53	56	54	57
**5′ UTR**	63	56	50	51	55
**3′ UTR**	65	60	69	51	69
**Intergenic**	303	179	238	173	242

We next analyzed RNA-seq datasets comprising both coding genes and predicted noncoding sRNA loci from the Bmb9393 strain together with previously published datasets of US strains (Table 18, available as supplementary data at *Bioinformatics Advances* online). To identify orthologous genes across all strains, we implemented a homology-based workflow using ProteinOrtho to infer shared and strain-specific gene sets and transfer functional annotations. Regarding coding sequences (CDSs) in *S. aureus* Bmb9393, we retrieved 2934 CDSs, and the compared strains contained 2706 (MW2), 2736 (N315), 2802 (MRSA252), and 2857 (LAC/NRS385) CDSs (see Tables 6–11, available as supplementary data at *Bioinformatics Advances* online). ProteinOrtho identified 2330 orthologous groups shared by all strains, with Bmb9393 exhibiting the largest number of unique genes (*n* = 212). Ortholog assignments can be cross-checked in Tables 6–11, available as supplementary data at *Bioinformatics Advances* online, where we list, for each gene ID row, the corresponding locus tags from all strains whenever orthology is established. Overall, 80.42% of gene groups were conserved across strains belonging to ST239 and US-lineage backgrounds.

Having established orthology relationships among coding genes, we next evaluated conservation of the predicted sRNA repertoire across the six strains. Our RNA-seq analysis detected 291 sRNAs common to all strains, with sequence identity typically in the 70%–80% range (Fig. 1, available as supplementary data at *Bioinformatics Advances* online). To define the presence of an sRNA in our data, we required a mean abundance of at least 10 reads across biological replicates after TMM and RPKM normalization. This threshold was chosen based on the principle that low read counts are often associated with higher variability and reduced confidence in gene expression measurements, particularly for sRNAs. The number of sRNAs detected varied across strains, with Bmb9393 showing the highest count (512), followed by N315 (443), LAC (437), MW2 (433), NRS385 (406), and MRSA252 (404) (Fig. 1, available as supplementary data at *Bioinformatics Advances* online).

### 3.2 Differential expression of coding genes and sRNAs between biofilm and planktonic conditions

The differential-expression analyses described below correspond to the primary transcriptomic data used as input for the subsequent integrative prioritization framework. Our analysis of sRNA differential expression between biofilm and planktonic lifestyles highlights several state-specific sRNAs, consistent with context-dependent regulatory functions. We identified 70 differentially expressed (DE) sRNAs that were upregulated in common to all five strains, but this number decreases to 10 when Bmb9393 is included (Fig. 2, available as supplementary data at *Bioinformatics Advances* online). Notably, we identified several sRNAs that strongly correlate with biofilm growth when comparing these two sets. Similarly, the set of downregulated sRNAs in biofilm includes 39 shared among the five strains excluding Bmb9393, but this number drops to just 4 when Bmb9393 is included in the analysis (Fig. 2, available as supplementary data at *Bioinformatics Advances* online).

The heatmap in Fig. 2 shows the sRNAs consistently upregulated or downregulated across all strains. Specifically, 10 sRNAs were consistently upregulated, including rsaH, sRNA160, sRNA4, Teg13, sprG3, sprF3, artR, Sau6873, Sau92, and Teg38as, while four sRNAs were consistently downregulated, namely Teg112, RsaX05, sRNA47, and Sau14. This consistent expression suggests that specific sRNAs may play conserved regulatory roles in biofilm and planktonic conditions across the *S. aureus* lineages studied.

**Figure 2:**
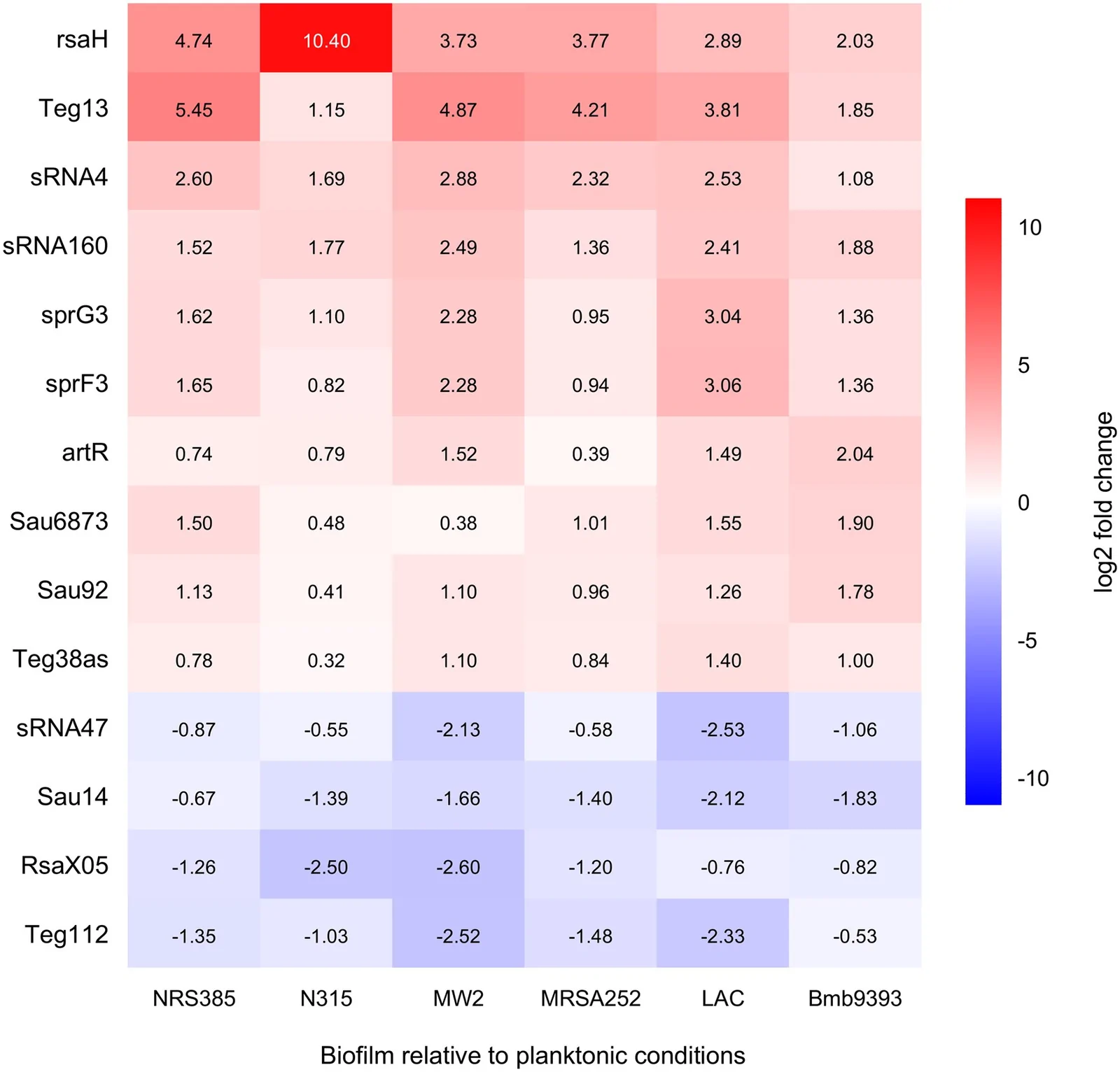
Heatmap of sRNAs consistently up- and downregulated across all strains. Fourteen sRNAs are highlighted. Colors and cell values indicate log2 fold changes for biofilm relative to planktonic conditions, taken from Tables 6–11, available as supplementary data at *Bioinformatics Advances* online. Those upregulated include rsaH, Teg13, sRNA4, sRNA160, sprG3, sprF3, artR, Sau6873, Sau92, and Teg38as, while downregulated sRNAs comprise sRNA47, Sau14, RsaX05, and Teg112. “sRNAs” names according to the most common name entry in the SRD (https://srd.genouest.org).

A comprehensive evaluation of upregulated sRNAs under biofilm conditions identified several candidates for deeper investigation. Among 70 sRNAs commonly upregulated across five public *S.aureus* strains, notable examples include rsaH, artR, RsaOV, Teg23, sprF3, sprG3, Teg13, and Sau6667 (Tables 6–11, available as supplementary data at *Bioinformatics Advances* online). A study by Burke (2018) showed that mutations in these sRNAs altered biofilm phenotypes in a USA300 strain. Notably, rsaH, artR, Teg13, sprG3, and sprF3 were also upregulated in Bmb9393, reinforcing their potential role in biofilm formation and regulation across *S.aureus* lineages (Table 6, available as supplementary data at *Bioinformatics Advances* online).

The upregulated sRNAs, sprG3, and sprF3 were consistently identified across all analyzed *S.aureus* strains and are particularly notable. These sRNAs are transcribed from genes encoding a toxin–antitoxin (TA) system, in which the antitoxin prevents protein synthesis in *trans*-acting cells (Chlebicka *et al.* 2021, Sarpong and Murphy 2021). The SprG3/SprF3 TA systems are implicated in promoting persister cell formation under stress conditions, enhancing biofilm resilience through bacteriostatic activity rather than inducing cell death (Riffaud *et al.* 2019).

The sRNA artR was consistently upregulated and was reported to modulate hla expression by repressing *sarT* mRNA, both directly and through RNAIII-mediated degradation. Previous studies showed that *agrA* negatively regulates artR (Schmidt *et al.* 2001, Xue *et al.* 2014); this relationship was reflected in most strains analyzed, except for Bmb9393 and N315, where *agrA* was upregulated under biofilm conditions. The *sarT* expression patterns also varied: it was generally downregulated, whereas artR was upregulated, with slight increases in N315 and NRS385. These results suggest artR may regulate additional targets. Altogether, these findings underscore the complex, strain-specific roles of sRNA-mediated regulation in *S. aureus* biofilm formation.

Prior work proposed that Sau41 represses the Agr circuit by inhibiting RNAIII, providing negative feedback that limits hypervirulence (Yu *et al.* 2023). In the present study, we interpret the Sau41–RNAIII relationship as a working, context-dependent model in which Sau41 may function as a competitive “sponge” or decoy-like RNA that modulates the fraction of RNAIII available to activate *hla* translation, thereby influencing α-hemolysin output in biofilm. This interpretation is compatible with a regulatory configuration that could help maintain a stable, nondispersive biofilm architecture by buffering toxin levels rather than fully suppressing Agr signaling. In the canonical pathway, RNAIII activates *hla* translation by binding to its 5′UTR, introducing structural rearrangements that expose the SD region to initiate translation (Morfeldt *et al.* 1995). Within this framework, increased Sau41 abundance could reduce the free pool of RNAIII available for *hla* activation, potentially attenuating α-hemolysin production and shaping biofilm-associated virulence output.

This scenario is most strongly supported in Bmb9393, known for producing high levels of biofilm (Costa *et al.* 2023), in which *hla* (SABB_RS07920; baseMean 154) and RNAIII (baseMean 7114) display no significant log2FC between biofilm and planktonic conditions, whereas Sau41 increases (baseMean 3670; log2FC 1.1) (see Table 6, available as supplementary data at *Bioinformatics Advances* online). Although *hla* and RNAIII do not pass the statistical threshold via DEG analysis, the ΔΔCt qRT-PCR profiles recapitulate this pattern. Relative to RNAIII, Sau41 is present at about half the level and *hla* at about twice the level. These expression patterns provide a basis for investigating a context-dependent relationship among Sau41, RNAIII, and *hla* in Bmb9393. The qRT-PCR measurements describe changes in each transcript across growth conditions; testing the proposed competition-based mechanism would require direct measurements of RNA–RNA interactions and relative molecular abundance. Given Hla’s capacity to lyse immune cells, including macrophages (Scherr *et al.* 2015), such tuning could help balance virulence output with maintenance of a cohesive biofilm structure. In contrast, in the US-lineage strains, Sau41, RNAIII, and *hla* displayed heterogeneous expression patterns that were not consistent with the same stoichiometric model. Accordingly, we interpret the proposed sponge-like interaction as context-dependent and most clearly supported in Bmb9393. Another important sRNA, rsaA, is exclusively upregulated in the US strains (Tables 7–11, available as supplementary data at *Bioinformatics Advances* online) and is a well-characterized regulator of biofilm formation (Romilly *et al.* 2014). Romilly *et al.* (2014) demonstrated that rsaA binds to *mgrA* mRNA, blocking the SD sequence and inhibiting its translation, thereby promoting an alternative, *ica*-independent biofilm pathway. In our data, rsaA was consistently upregulated across all US strains but not significantly altered in Bmb9393 under biofilm conditions (Tables 6–11, available as supplementary data at *Bioinformatics Advances* online). The MgrA protein generally mirrors the *agr* program and functions as a negative regulator of biofilm, repressing adhesins and autolysis while activating extracellular enzymes that degrade matrix components (Costa *et al.* 2023). Consequently, *mgrA* downregulation is expected to favor the biofilm state. Consistent with this, Bmb9393 exhibited the most substantial decrease in *mgrA* expression (log2FC −1.42) despite the absence of rsaA upregulation, whereas USA500 (NRS385) showed a moderate decline (log2FC −0.92) alongside rsaA induction (Tables 6–11, available as supplementary data at *Bioinformatics Advances* online). In contrast, Bmb9393 appears to downshift *mgrA* via alternative regulatory inputs, such as the WalKR two-component system (*walK* was upregulated in Bmb9393; see Δ  ΔCt in Section 3.9), which has been suggested to control *mgrA* expression negatively (Guo *et al.* 2025). Both scenarios converge on reduced MgrA output, which relaxes repression of adhesins and autolysis and limits the activity of matrix-degrading enzymes, thereby reinforcing biofilm maintenance.

### 3.3 Coexpression modules and hub transcripts

The network analyses described below are based exclusively on transcript coexpression and therefore precede the interaction-prioritization framework introduced in the following section. The WGCNA co-expression analysis identified 23 modules of coexpressed transcripts. Seven modules showed significant module-trait correlations with the biofilm condition (*P* ≤ .05), while others were associated with the planktonic state (Fig. 3). Table 12, available as supplementary data at *Bioinformatics Advances* online, provides a comprehensive compendium of module assignments for all CDSs and sRNA loci across the studied strains, enabling cross-lineage comparisons and revealing both shared and strain-specific signatures. In WGCNA, modules group transcripts displaying similar expression profiles across samples, whereas hub transcripts correspond to the most highly connected nodes within each module and often represent key components of the underlying biological processes. Table 13, available as supplementary data at *Bioinformatics Advances* online, provides details of the hub assignments for each module associated with the planktonic or biofilm state. These modules represent coordinated transcriptional programs in which genes with correlated expression are frequently coregulated by shared transcription factors or sRNAs. Therefore, these modules can represent distinct physiological states or adaptive responses, providing a functional framework for interpreting the regulatory role of the sRNAs identified as central nodes in the network.

**Figure 3:**
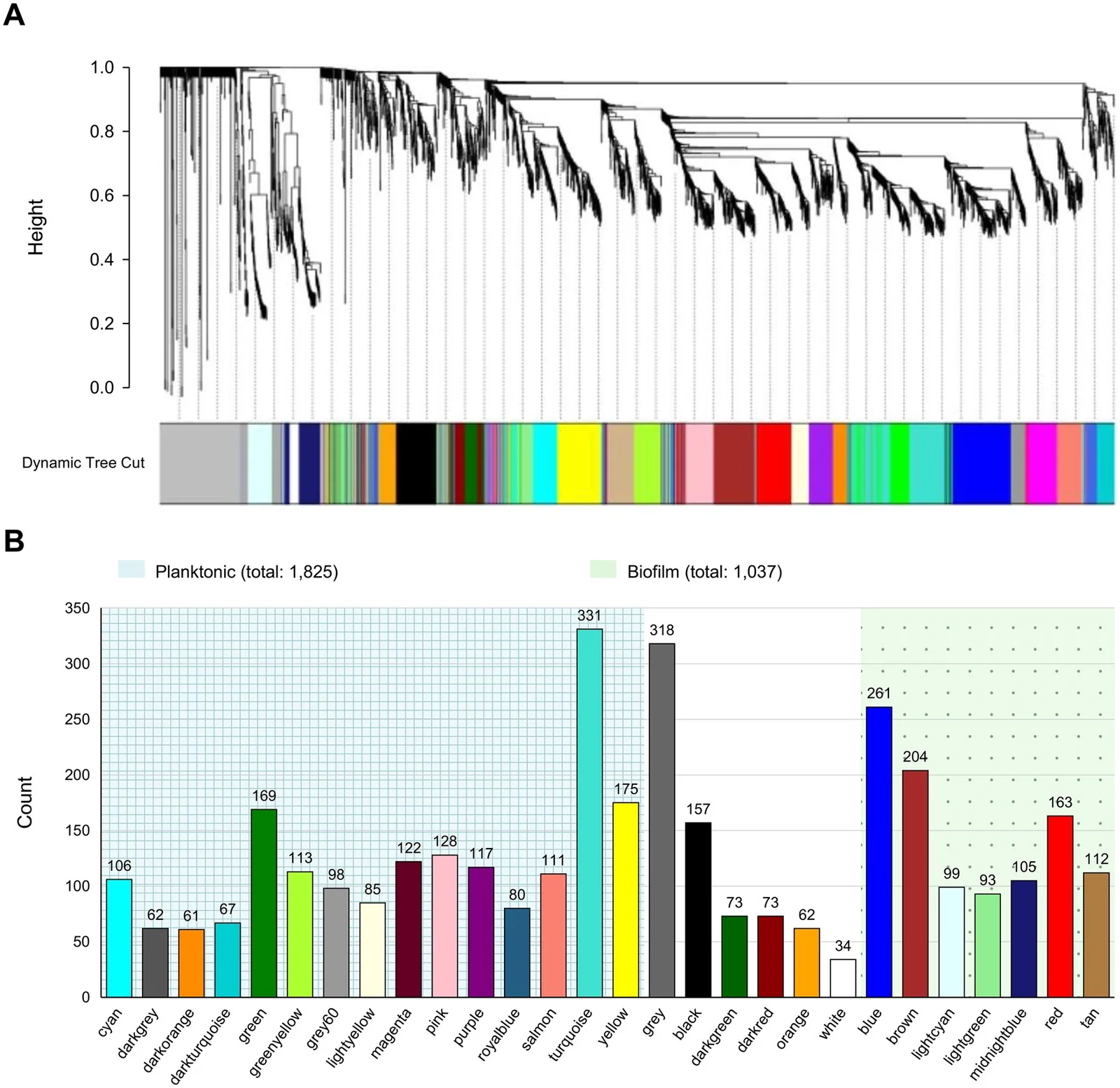
Module composition under planktonic and biofilm conditions. (A) The upper panel shows the hierarchical clustering dendrogram generated by WGCNA, in which genes are grouped into modules according to shared co-expression patterns. Each module is represented by a unique color corresponding to a cluster of highly coexpressed genes. (B) The lower panel summarizes module composition, with bar heights indicating module size. Modules significantly correlated (*P* ≤ .05) with the planktonic condition (1825 genes) are indicated by fine checkered shading, whereas modules associated with the biofilm condition (1037 genes) are indicated by dotted shading. Modules with white shading were not significantly associated.

Gene Ontology enrichment yielded biologically interpretable insights for 17 modules (Table 14, available as supplementary data at *Bioinformatics Advances* online). Among these, the brown module was the principal biofilm-associated cluster, enriched for metabolic adaptation pathways, including urease activity (*ureA*, *ureB*, and *ureC*) and nitrogen metabolism (*glnA* and *narT*), consistent with Tomlinson *et al.* (2021). These pathways contribute to pH regulation and survival in nutrient-limited, anaerobic biofilm environments (Park *et al.* 2020, Vudhya Gowrisankar *et al.* 2021). The brown module’s hub, SABB_RS04005, encodes a diacylglycerol kinase (DgkB) that recycles diacylglycerol for lipoteichoic acid (LTA) biosynthesis, contributing to cell-envelope homeostasis in *S. aureus* (Miller *et al.* 2008). Perturbations of LTA synthesis, processing, and related signaling pathways have been shown to influence biofilm phenotypes (Ahn *et al.* 2018). Accordingly, the prediction of DgkB as a highly connected node in this biofilm-associated module is consistent with known envelope controls of biofilm phenotype.

The tan module, another biofilm-associated cluster, was enriched with nucleotide metabolism genes (*guaA*, *guaB*, and ppGpp pathways; Giammarinaro *et al.* 2022), which were downregulated in US strains but upregulated in Bmb9393 (Tables 6–11, available as supplementary data at *Bioinformatics Advances* online), suggesting strain-specific adaptations (Giammarinaro *et al.* 2022). The tan hub gene, SABB_RS03985, encodes an ABC transporter linked to antioxidant defenses via L-ergothioneine uptake (Zhang *et al.* 2022b), with potential roles in ROS/RNS resistance during infection.

Notably, the three components of the Sau41–RNAIII–*hla* axis were localized to modules significantly associated with the biofilm state. Sau41 and RNAIII were coassigned to the tan module, whereas *hla* mapped to the blue module (Table 12, available as supplementary data at *Bioinformatics Advances* online).

Additional hub genes from the modules include SABB_RS14820, an anaerobic ribonucleotide reductase activator involved in low-oxygen adaptation (Ollagnier *et al.* 1997, Ewels *et al.* 2020), and SABB_RS04360, encoding ClfA, an MSCRAMM adhesin critical for host colonization, immune evasion, and endocarditis initiation (Palmqvist *et al.* 2004).

Two particularly notable findings involve sRNAs identified as potential gene hubs in two planktonic-associated modules: sbrC and Teg20. Notably, no targets have yet been validated for these sRNAs. To our knowledge, no previous study has associated Teg20 with either the biofilm or planktonic lifestyle, making this the first report of such an association. Teg20 is characterized as a *cis*-acting regulator functioning as a riboswitch for *glmS* (Beaume *et al.* 2010, Howden *et al.* 2013). The *glmS* gene encodes glucosamine-6-phosphate synthase, which catalyzes the conversion of fructose-6-phosphate and glutamine to glucosamine-6-phosphate. Its regulatory ribozyme responds to glucosamine-6-phosphate by promoting self-cleavage of the RNA (Ferré-D’Amaré 2010). Beyond this metabolic and regulatory context, studies using a *glmS* mutant have implicated the gene in *S. aureus* biofilm formation, particularly in the response to advanced glycation end products (AGEs). These studies describe enhanced biofilm formation facilitated by promoting eDNA release through the upregulation of *sigB* in *S. aureus*. Notably, the *glmS* mutant exhibits several phenotypic changes, including pigment deficiency, reduced hemolytic capacity, downregulation of *hla* and *hld* expression, and the formation of thinner, sparser biofilms.

Furthermore, these findings indicate that, in the mutant strain, *sigB* and biofilm formation are no longer responsive to AGEs (Ni *et al.* 2024). Our DEG analysis showed that *glmS* expression was downregulated under biofilm conditions in most US strains, which correlates with reduced *hla* expression reported in the previous section (Tables 7–11, available as supplementary data at *Bioinformatics Advances* online). Notably, *glmS* is part of the green-yellow module, which is associated with planktonic conditions. At the same time, Teg20 showed heterogeneous expression across strains, suggesting that the role of advanced glycation end products in promoting biofilm formation may involve this pathway in a strain-specific manner.

Our findings regarding sbrC showed that this sRNA was significantly downregulated across multiple US strains in biofilm: MRSA252 (log FC −2.4), NRS385 (log FC −2.9), N315 2 2 (log FC −1.2), LAC (log FC −3.3), and MW2 (log FC −3.3) (Tables 7–11, available as supplementary data at *Bioinformatics Advances* online). Furthermore, sbrC is part of the SigB regulon (Nielsen *et al.* 2011), a critical regulator of stress responses and cellular adaptation. The upregulation of sbrC in planktonic cells suggests its involvement in promoting or maintaining this lifestyle, potentially by regulating factors that inhibit biofilm formation. Its association with the SigB regulon aligns with its proposed role in stress adaptation, a crucial process for survival in the planktonic state. Given its regulatory association with SigB, these findings suggest that sbrC may modulate biofilm dynamics in *S. aureus*.

### 3.4 Computational prediction and calibration of the sequence-based prediction layer

To assess how sequence-based score thresholds affected candidate retention, we compared three alternative threshold schemes (Table 2) applied to the original prediction outputs generated by IntaRNA, RNAplex, TargetRNA3, and sRNARFTarget. These schemes comprised a calibrated tool-specific configuration, used for the main analysis, together with restrictive common-threshold and highly restrictive common-threshold configurations (Fig. 4).

**Figure 4:**
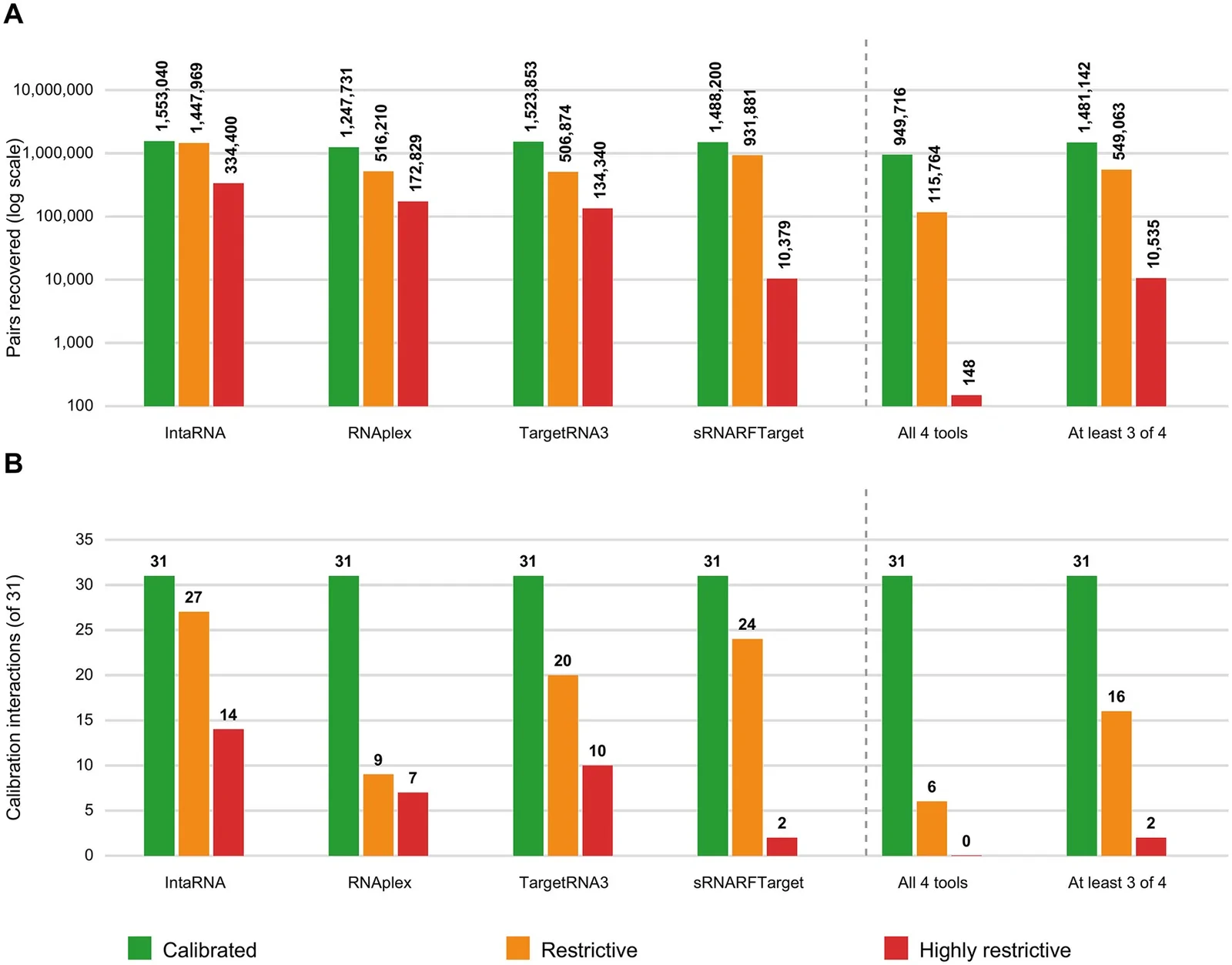
Retention of candidate and calibration sRNA–mRNA interactions across threshold schemes. Effect of three threshold schemes on the retention of predicted and literature-curated sRNA–mRNA interactions across four prediction tools (IntaRNA, RNAplex, TargetRNA3, and sRNARFTarget) and two consensus-support criteria (supported by all four tools or by at least three of four tools). (A) All predicted candidate sRNA-mRNA pairs (*n* = 1 731 060; log scale). (B) The 31 literature-curated calibration interactions (Table 20, available as supplementary data at *Bioinformatics Advances* online). Bars represent the calibrated tool-specific (green), restrictive common-threshold (orange), and highly restrictive common-threshold (red) schemes. The calibrated cutoffs (sRNARFTarget probability ≥ .45, IntaRNA *P*-value ≤ .41, RNAplex *P*-value ≤ .76, and TargetRNA3 *P*-value ≤ .89) were defined to retain all 31 calibration interactions before downstream biological-context filtering. Their complete retention under this scheme is therefore expected by construction and does not represent independent validation.

To define the calibrated thresholds, we compiled a reference set of 31 literature-curated sRNA–mRNA interactions that could be mapped to the Bmb9393 locus annotation (Table 20, available as supplementary data at *Bioinformatics Advances* online). We used these interactions to establish tool-specific cutoffs that retained the complete calibration set at the sequence-based prediction stage, before downstream biological-context filtering. They did not constitute an independent validation dataset.

Retention of the 31 curated interactions was quantified for each prediction tool individually and under two consensus-support criteria, requiring support by either all four prediction tools or at least three of four tools. The three-of-four criterion was included for comparison; the downstream prioritization workflow required support by all four tools.

Progressively more stringent threshold schemes reduced the number of retained calibration interactions (Fig. 4). Each prediction tool retained all 31 interactions under both consensus criteria, as expected from the threshold-selection procedure. The restrictive common-threshold scheme retained 6 of the 31 interactions under the four-tool criterion and 16 under the three-of-four criterion. Under the highly restrictive common-threshold scheme, no calibration interactions satisfied the four-tool criterion, whereas two satisfied the three-of-four criterion.

These comparisons describe how threshold stringency affected retention of the calibration interactions. Their retention under the calibrated scheme reflects its design objective: preserving the literature-curated reference set at the initial sequence-based prediction stage. Candidate interactions passing this stage were subsequently prioritized using differential-expression and coexpression criteria.

### 3.5 Stepwise attrition across the integrative prioritization framework

To understand how each layer of evidence contributed to the final prioritization, we quantified the number of candidate sRNA–mRNA interactions retained after every step of the workflow under the three threshold schemes evaluated in this study (Fig. 5; Table 2). In parallel, we followed the behavior of the 31 literature-curated interactions throughout the same filtering process, allowing us to identify at which stage these experimentally supported interactions were retained or excluded.

**Figure 5:**
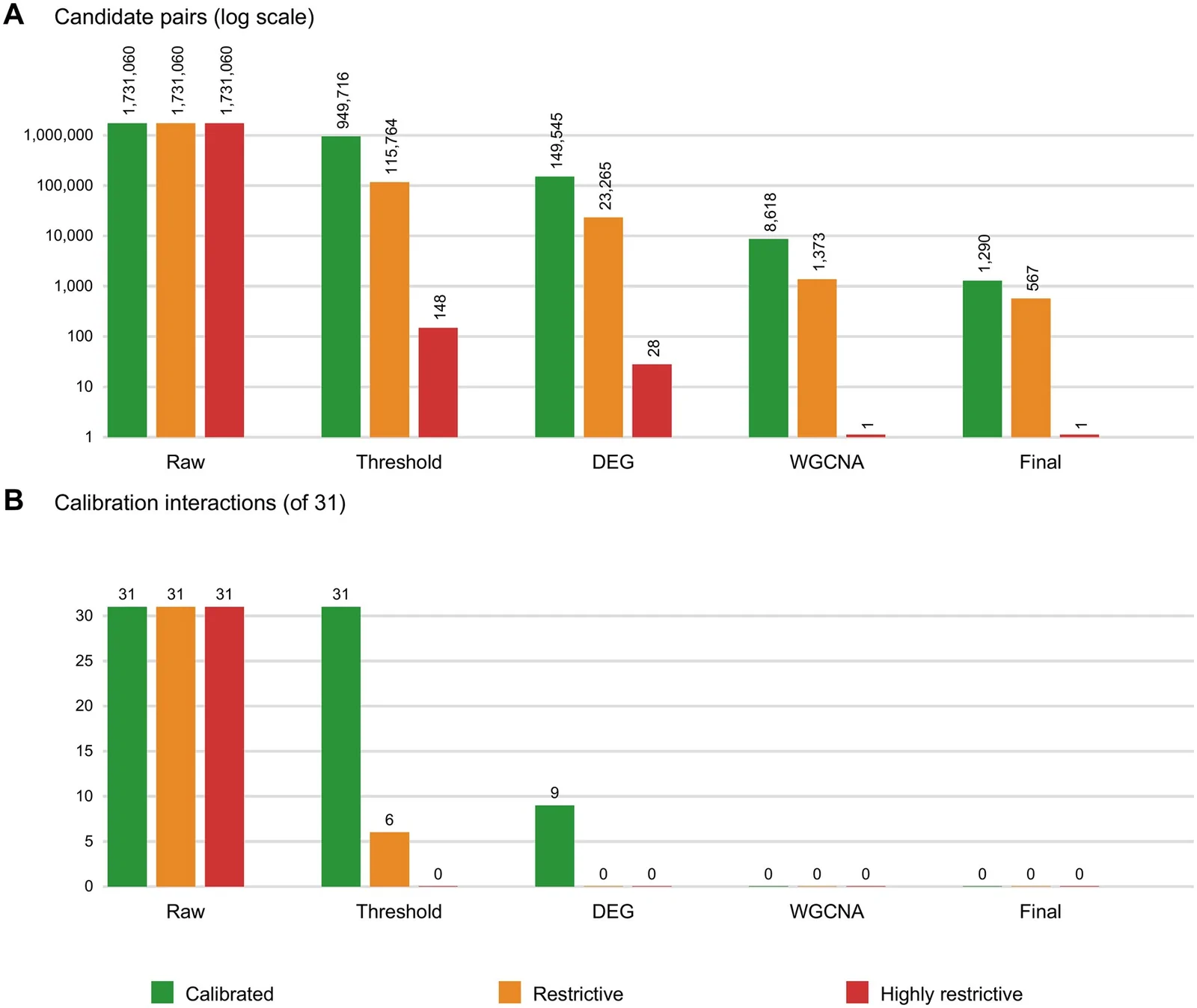
Stepwise filtering and prioritization of sRNA–mRNA interactions across three threshold schemes. Number of interactions retained after successive workflow steps: raw (all predicted candidate pairs), threshold (sequence-based score cutoffs with support from all four prediction tools), DEG (differential-expression presence consensus), WGCNA (same-module coassignment plus presence of a connecting edge in the network exported with a threshold of 0.1), and final (target-level prioritization by selecting the highest-weight sRNA partner per target). Counts are cumulative: each step operates on the candidates retained at the preceding step. (A) Candidate-pair attrition for all predicted interactions (log scale). (B) Retention of the 31 literature-curated calibration interactions (out of 31). Bars represent the calibrated tool-specific (green), restrictive common-threshold (orange), and highly restrictive common-threshold (red) schemes. The differential-expression presence-consensus criterion was applied to the sRNA component of each pair (*P*_adj_  ≤ .05 and baseMean ≥ 10 in at least five of the six strain-specific comparisons). We applied no additional downstream minimum edge-weight cutoff.

The three schemes exhibited the same pattern of progressive candidate reduction, differing only in the sequence-based prediction thresholds applied at the initial filtering step. As expected, the calibrated tool-specific configuration retained the largest number of candidate interactions throughout the workflow, whereas progressively more stringent threshold schemes produced progressively smaller candidate sets (Fig. 4A; Table 2). Under the calibrated configuration, the initial 1 731 060 predicted interactions were reduced to 949 716 after applying the sequence-based thresholds, to 149 545 after the differential-expression presence-consensus filter, and to 8618 after the WGCNA same-module and network-edge requirements. The final target-level prioritization step selected, for each target gene, the candidate sRNA with the highest coexpression edge weight, yielding a final set of 1290 prioritized interactions. The same workflow generated final sets of 567 and 1 interaction under the restrictive and highly restrictive threshold schemes, respectively.

All 31 literature-curated calibration interactions were retained after applying the calibrated sequence-based thresholds, as expected from the calibration procedure (Fig. 5B). Increasing threshold stringency reduced retention to six interactions under the restrictive scheme and none under the highly restrictive scheme, consistent with the four-tool consensus results shown in Fig. 4. In the calibrated workflow, nine interactions remained after the differential-expression presence-consensus filter (sRNA *P*_adj_  ≤ .05 and baseMean ≥10 in at least five of the six strain-specific comparisons), and none remained after the WGCNA filtering step. Under the restrictive scheme, none of the six interactions retained at the prediction stage passed the subsequent differential-expression filter. Thus, no calibration interactions remained in the final prioritized sets, although they were excluded at different stages across the three schemes.

In the calibrated workflow, 8618 candidate sRNA–mRNA pairs satisfied the sequence-, expression-, and network-based requirements, including assignment of both partners to the same WGCNA module and the presence of their connecting edge in the network exported with a threshold of 0.1 (Table 2). No additional downstream minimum edge-weight cutoff was applied. The subsequent reduction to 1290 interactions resulted from target-level prioritization. When multiple candidate sRNAs were associated with the same target gene, only the interaction with the highest coexpression edge weight was retained.

These analyses document how sequence-based thresholds, biological-context requirements, and target-level partner selection successively narrowed the candidate interaction sets. The resulting pairs represent candidates prioritized using expression and coexpression evidence from the analyzed conditions for subsequent experimental investigation.

### 3.6 Overlap of the final prioritized interaction sets across threshold schemes

We compared the final interaction sets generated under the three threshold schemes using the Jaccard index at three levels: exact sRNA–mRNA interaction pairs, target mRNAs, and sRNAs (Fig. 6).

**Figure 6:**
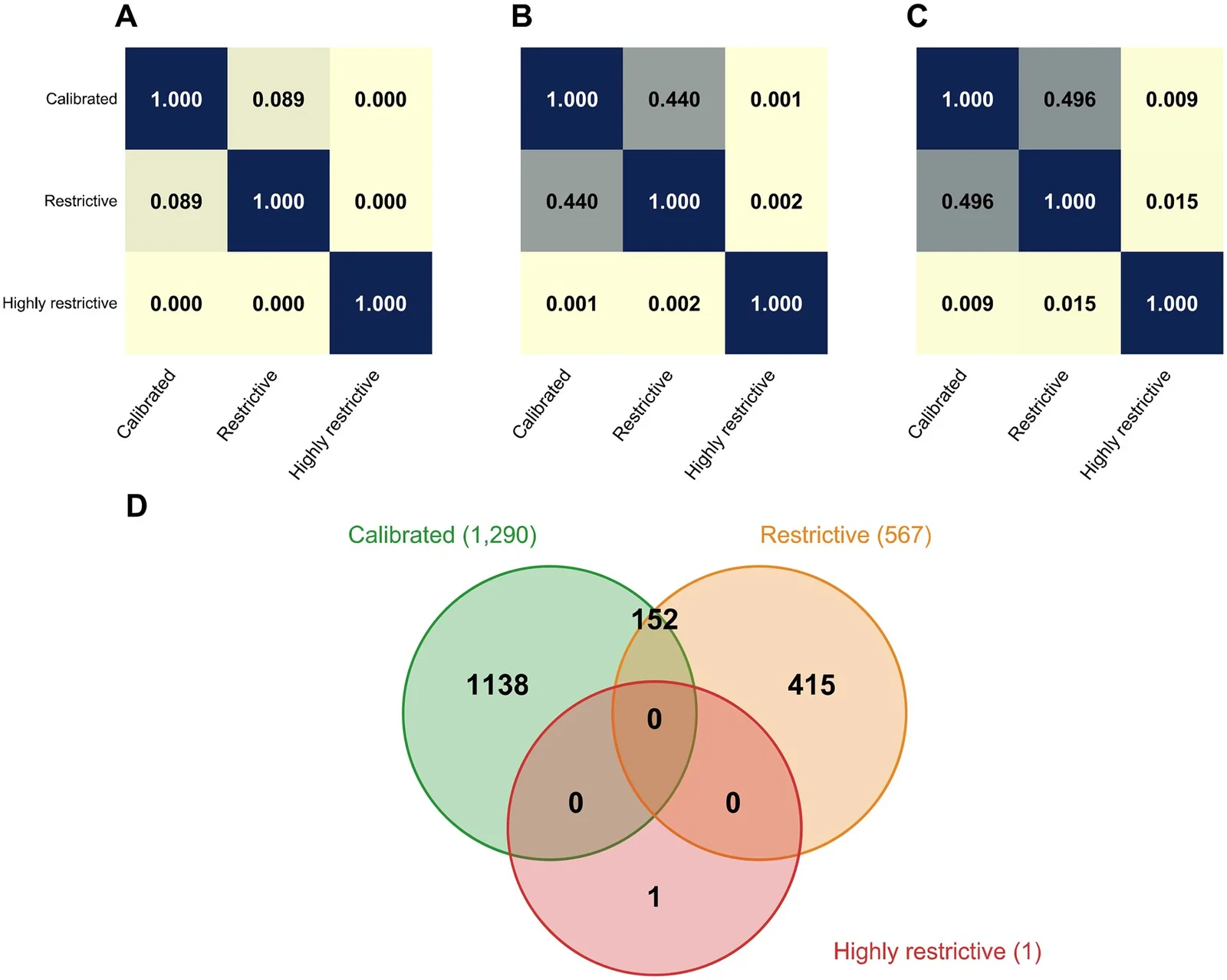
Overlap of prioritized sRNA–mRNA pairs, target mRNAs, and sRNAs across threshold schemes. Heatmaps show pairwise Jaccard indices computed for (A) exact sRNA-mRNA interaction pairs, (B) target mRNAs, and (C) sRNAs for the calibrated, restrictive, and highly restrictive schemes. (D) The Venn diagram shows the number of exact final interaction pairs unique to, or shared between, the calibrated (*n* = 1290), restrictive (*n* = 567), and highly restrictive (*n* = 1) schemes.

The calibrated and restrictive schemes shared 152 exact interaction pairs (Jaccard = 0.089), with 1138 pairs unique to the calibrated set and 415 unique to the restrictive set. All 567 target mRNAs retained under the restrictive scheme were also represented in the calibrated set, but only 152 retained the same selected sRNA partner; the remaining 415 were associated with different partners. Overlap was therefore greater at the target-mRNA and sRNA levels, with Jaccard indices of 0.440 and 0.496, respectively (Fig. 6).

Target-set nesting was expected because the restrictive candidate pool was a subset of the calibrated pool and both underwent identical downstream filtering. This nesting reflects the workflow design, while the frequent changes in selected sRNA partners indicate substantial threshold sensitivity in individual sRNA–mRNA assignments.

The highly restrictive scheme retained a single interaction pair that was absent from both larger final interaction sets, although its target mRNA and sRNA were individually represented in both (Fig. 6). The low target-mRNA and sRNA Jaccard indices reflect the pronounced differences in set size.

### 3.7 Biological interpretation of prioritized sRNA–mRNA interactions

Having established the calibration strategy, quantified candidate attrition across the integrative workflow, and compared the final prioritized interaction sets across alternative threshold schemes, we next examined the biological interpretation of the prioritized candidates (Table 15, available as supplementary data at *Bioinformatics Advances* online). We highlight representative sRNA–mRNA pairs that illustrate distinct biological processes associated with biofilm formation, virulence, metabolism, and cell-envelope homeostasis.

Despite the growing number of annotated sRNAs in *S. aureus*, the biological functions of most remain unknown, and even fewer have experimentally characterized regulatory targets. Consequently, our discussion focuses on representative interactions emerging from the integrative prioritization framework and on the biological functions of their associated target genes, which provide testable hypotheses for future experimental validation. Table 4 summarizes 15 selected representative interactions from the final prioritized dataset.

**Table 4:** High-priority sRNA–mRNA pairs identified by the framework.^a^

sRNA	Gene or product	Bmb9393 ID
**sRNA255**	*sceD*	SABB_RS11865
**SprA1**	*ebpS*	SABB_RS06145
**sRNA235**	*ltaS*	SABB_RS03960
**Sau6356**	*srtA*	SABB_RS14235
**sprG3**	*sspA*	SABB_RS05325
**Teg65**	*sspB*	SABB_RS05320
**SprA1**	*hla*	SABB_RS07920
**sRNA4**	*clpP*	SABB_RS04255
**Teg13**	*fmtA*	SABB_RS05370
**Teg48**	YceI family protein	SABB_RS15215
**artR**	*adhP*	SABB_RS03360
**Sau6356**	*guaA*	SABB_RS02115
**sRNA4**	*walR*	SABB_RS00100
**sRNA259**	*nrdG*	SABB_RS14820
**srn_2058**	*clfA*	SABB_RS04360

a sRNA names follow the most common name entry in the Staphylococcal Regulatory RNAs Database (https://srd.genouest.org); srn_2058 follows Bronsard et al. (2017).

Among the prioritized interactions, the artR–*adhP* (*adhP*, SABB_RS03360) emerged as an interesting candidate linking sRNA regulation to metabolic adaptation and was also upregulated under biofilm conditions (Table 15, available as supplementary data at *Bioinformatics Advances* online). Interestingly, *adhP* was also significantly upregulated in the biofilm of *Stenotrophomonas maltophilia*, as reported by Alio *et al.* (2020). In *Escherichia coli*, *adhP* has been described as an ethanol-inducible dehydrogenase (Thomas *et al.* 2013). Alio *et al.* (2020) proposed that *adhP* likely contributes to the production of propanol or other short-chain alcohols under biofilm conditions (Alio *et al.* 2020). However, its role in biofilm development or maintenance remains unexplored. Further analysis of *adhP* under the biofilm conditions of *S. aureus* Bmb9393 could provide valuable insights into its functional contributions to biofilm formation and dynamics.

Our network analysis also identified high-priority interactions between sRNAs and hub coding genes within biofilm-associated WGCNA modules (Table 13, available as supplementary data at *Bioinformatics Advances* online), revealing module-specific coupling of adhesion, metabolism, lipid signaling, and transport to sRNA-mediated regulation. In the blue module, *clfA* (SABB_RS04360) is predicted to be targeted by srn_2058; and in the red module, sRNA259 aligns with *nrdG* mRNA (SABB_RS14820; Class III anaerobic ribonucleotide reductase). These associations, retained after cross-tool, coexpression, and expression-consistency filters, underscore coordinated sRNA regulation across biofilm-relevant functional hubs. Future experimental validation of these interactions will be essential to uncover the precise mechanisms by which sRNAs influence biofilm dynamics in *S. aureus*.

The prioritized interactions identified here provide a practical starting point for experimental validation. In practical terms, the most direct validation route would be to perturb the sRNA itself, such as through CRISPRi knockdown (Prezza *et al.* 2024) or overexpression (Bohn *et al.* 2010), and then quantify the impact on the predicted target at the transcript and, where possible, protein or reporter level, together with the corresponding phenotype. For example, candidate pairs linked to cell-wall remodeling (sRNA255-*sceD*) and envelope homeostasis (sRNA235-*ltaS* and sRNA4-*walR*) could be evaluated by combining sRNA perturbation with qRT-PCR, transcript half-life measurements, and autolysis or antibiotic-susceptibility assays (Roberts *et al.* 2006). Pairs involving adhesion- and biofilm-related targets, such as srn_2058-*clfA* or sRNA4-*walR*, could be followed by reporter assays and biofilm phenotyping (Han *et al.* 2024). In contrast, pairs involving virulence- or toxin-associated targets, such as SprA1-*hla* and Sau6356-*srtA*, could be assessed through toxin- or host-interaction readouts (Hachani *et al.* 2023). In this framework, direct RNA–RNA binding assays such as EMSA, or mechanistic assays involving RNase-dependent processing would be best positioned for second-step validation once functional dependence on the sRNA has been established. Although these experiments are beyond the scope of the present computational study, our framework is intended to prioritize the most promising candidates for such targeted validation.

### 3.8 Genomic context for constraining sRNA target prediction

The genetic location of sRNAs is critical in target prediction, especially for antisense sRNAs transcribed in the opposite direction of a CDS. In such cases, predicting the corresponding CDS as the target gene is straightforward due to their complementary orientation. This natural complementarity allows for perfect or near-perfect base pairing, which significantly enhances the likelihood of interaction, for example, between an antisense sRNA and its target gene (Georg and Hess 2011). Then, we annotated the Bmb9393 genomic context of each sRNA (e.g. intergenic, intragenic, untranslated-region-associated [UTR-associated], antisense) and the associated CDS in Table 15, available as supplementary data at *Bioinformatics Advances* online, indicating whether the sRNA is in the sense or antisense orientation.

Among the antisense sRNAs identified in Bmb9393, two were retained after the complete prioritization workflow: Sau6356 and sRNA4, whose predicted targets correspond to the complementary CDSs SABB_RS02115 and SABB_RS00100, respectively (Table 15, available as supplementary data at *Bioinformatics Advances* online).

A particularly interesting finding is the interaction between sRNA4 and *walR* (SABB_RS00100), both of which are coexpressed in the turquoise module associated with the planktonic state (Tables 12 and 13, available as supplementary data at *Bioinformatics Advances* online). Notably, sRNA4 was one of the 10 sRNAs consistently upregulated across all six strains analyzed in this study, including the Bmb9393 strain (Table 6, available as supplementary data at *Bioinformatics Advances* online, and confirmed by qRT-PCR, Fig. 7). This consistent expression underscores its potential regulatory importance and suggests a conserved role in biofilm-associated processes.

**Figure 7:**
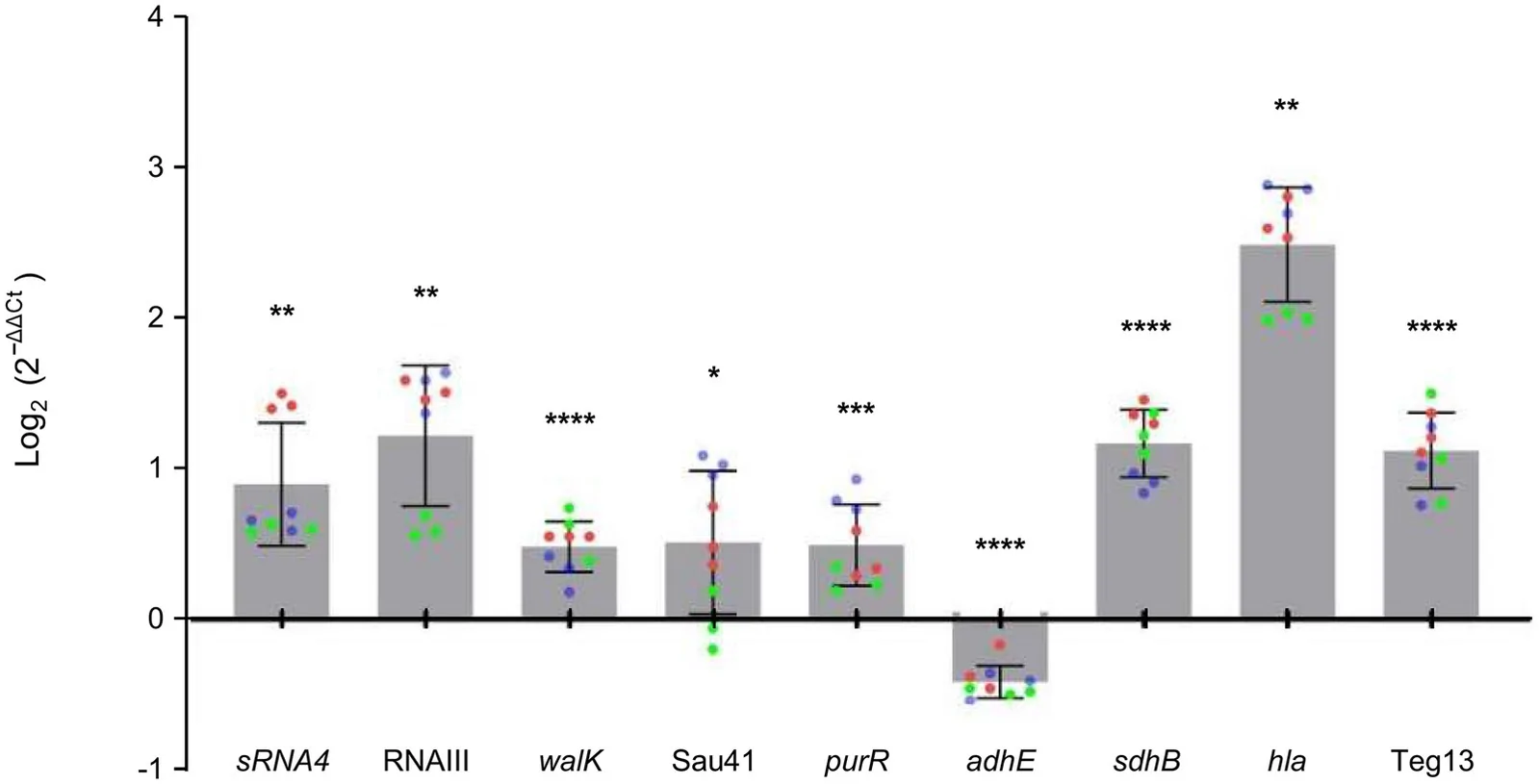
Real-Time qRT-PCR Validation of sRNA and mRNA Expression Under Biofilm Conditions (Bmb9393). qRT-PCR (Δ  ΔCt) comparison of biofilm versus planktonic conditions for nine loci in Bmb9393: *walK* (SABB_RS00105), sRNA4, RNAIII, Sau41, *hla*, *purR* (SABB_RS02705), *adhE* (SABB_RS00775), *sdhB* (SABB_RS08000), and Teg13. Relative expression was computed by Δ  ΔCt (see Methods section for calculation and sign convention). The direction of change (up/down in biofilm) matches the RNA-seq results for all loci (see Table 6, available as supplementary data at *Bioinformatics Advances* online). sRNAs are named according to the most common name entry in the SRD (https://srd.genouest.org). The colored dots on the bars denote the three technical replicates (same color) for each of the three biological replicates (different colors).

WalR, also upregulated in biofilm (Table 15, available as supplementary data at *Bioinformatics Advances* online), is the response regulator of the WalRK two-component system, a central controller of cell-wall metabolism in *S. aureus* (Fabret and Hoch 1998, Dubrac *et al.* 2007; Costa *et al.* 2023). This system is essential in Firmicutes and is highly conserved among low GC% Gram-positive bacteria, including pathogens such as *Streptococcus pneumoniae* and *S. aureus* (Takada and Yoshikawa 2018). Validated targets of the WalR regulon include genes involved in cell wall degradation and autolysis, such as *lytM*, *ssaA*, and *sceD*, as well as the gene encoding the adhesin EbpS, which is linked to virulence (Dubrac *et al.* 2007, Delauné *et al.* 2012). One of its most significant targets is *ltaS*, which encodes LTA. As mentioned before, LTA is crucial for cell-envelope homeostasis in *S. aureus* (Gründling and Schneewind 2007, Miller *et al.* 2008). The essentiality of *ltaS* has been demonstrated in multiple independent studies, with *ltaS* mutants found to be inviable (Chaudhuri *et al.* 2009, Santiago *et al.* 2015, Chen *et al.* 2017). This observed consistency suggests a direct connection between *ltaS* and the essential role of the WalRK system in biofilm (Delauné *et al.* 2012, Costa *et al.* 2023). In addition, *ltaS* overexpression enhances autolysis, hemolytic activity, and the transcription of virulence genes associated with host interaction, cytolysis, and immune evasion, partly through activation of the SaeSR TCS (Delauné *et al.* 2012).

Beyond its role in biofilm regulation, WalRK has also been associated with antimicrobial resistance, particularly vancomycin resistance. Strains with intermediate resistance to vancomycin frequently harbor mutations in the *walRK* promoter and operon genes (Hafer *et al.* 2012, Peng *et al.* 2016). Notably, *ltaS* is an appealing drug target because LTA is absent in eukaryotic cells (Gründling and Schneewind 2007).

In contrast to *Enterococcus faecalis*, where an antisense sRNA targeting *walR* reduces WalR and biofilm formation (Wu *et al.* 2021), the concordant increase in sRNA4 and *walR* in *S. aureus* suggests a stabilizing role for *walR*. We observed that sRNA4 (minus strand, nt 24 953–25 188) lies antisense to *walR* (plus strand, nt 24 903–25 604; SABB_RS00100), creating a CDS-internal overlap. Both transcripts are upregulated in biofilm, and qRT-PCR corroborates the upregulation of sRNA4. We hypothesize that this internal antisense pairing either occludes endonucleolytic sites and limits ribonuclease access or functions as a post-transcriptional fine-tuner under biofilm conditions to sustain *walR* when transcriptional input is high, both mechanisms consistent with elevated steady-state *walR* despite duplex formation. This is a compelling hypothesis for future experimental testing.

On the other hand, intragenic (sense) sRNAs are unlikely to base-pair with the mRNAs of their host genes because sense–sense pairing lacks the complementarity required for canonical sRNA–mRNA regulation. Accordingly, these sRNAs are often cotranscribed with their hosts and tend to mirror their expression profiles. Likewise, sRNAs positioned in 5′ or 3′ UTRs (distinct from bona fide riboswitches) do not inherently have a higher probability of targeting their cognate genes (Wagner and Romby 2015).

Among the intragenic sRNAs, Teg13 (encoded within SABB_RS11020) is 1 of 10 consistently upregulated across all 6 strains (Tables 6–11, available as supplementary data at *Bioinformatics Advances* online), and as mentioned previously, its gene deletion causes a defective biofilm phenotype. After applying our multistage filters, we retained 55 candidate Teg13 target pairs, all of which grouped into the yellow (planktonic-associated) module (Table 15, available as supplementary data at *Bioinformatics Advances* online). Notably, Teg13 and its host gene SABB_RS11020 were coassigned to the yellow module (i.e. coexpressed; Table 12, available as supplementary data at *Bioinformatics Advances* online). However, our pipeline did not predict a Teg13–SABB_RS11020 interaction, consistent with expectations for intragenic (sense) sRNAs.

Among Teg13’s predicted targets, *fmtA*, a well-characterized modulator of methicillin resistance in *S. aureus*, is especially notable. Analysis of the *fmtA* promoter demonstrated that the transcriptional level of *fmtA* increased when the cells were exposed to β-lactam antibiotics (Komatsuzawa *et al.* 1999). In our data, *fmtA* is downregulated in biofilm across the US-lineage strains (but unchanged in Bmb9393). In comparison, Teg13 is upregulated in biofilm and coassigned with its host gene to the yellow module. This pattern supports the hypothesis that Teg13 contributes to post-transcriptional repression of *fmtA* in a lineage-dependent manner. Previous studies have also demonstrated that *fmtA* expression is regulated by SarA, a critical regulator involved in biofilm formation (Komatsuzawa *et al.* 1999). SarA promotes biofilm development by negatively regulating protease and nuclease activities, essential for biofilm maintenance and stability (Abdelhady *et al.* 2014, Atwood *et al.* 2015, Liu *et al.* 2024). Biologically, decreasing FmtA during biofilm growth could sensitize cells to β-lactams, a potentially favorable therapeutic outcome. However, it may also increase autolysis and eDNA release, effects that reinforce matrix integrity and stabilize the biofilm, thereby complicating eradication. These observations argue for context-specific strategies: if Teg13-mediated repression of *fmtA* is confirmed, combining Teg13 overexpression with β-lactams and matrix-disrupting adjuncts (e.g. DNase or antibiofilm agents) could enhance β-lactam susceptibility while limiting biofilm matrix reinforcement.

Finally, as an example of target prediction involving a UTR-associated sRNA, we highlight Teg48, which originates from the 5′ UTR of SABB_RS03420 (Tables 1 and 15, available as supplementary data at *Bioinformatics Advances* online). After the complete prioritization workflow, Teg48 remained associated with the YceI family protein (SABB_RS15215), whereas the additional candidate interactions identified during the initial prediction stage were not retained after the biological-context filters.

### 3.9 qRT-PCR validation of sRNA and coding-gene expression under biofilm conditions

To orthogonally validate our RNA-seq results, we quantified transcript levels by qRT-PCR (ΔΔCt) for nine loci for strain Bmb9393: *walK* (SABB_RS00105), sRNA4, RNAIII, Sau41, *hla*, *purR* (SABB_RS02705), *adhE* (SABB_RS00775), *sdhB* (SABB_RS08000), and Teg13, comparing biofilm with planktonic conditions. The ΔΔCt profiles recapitulated the direction of change (upregulation versus downregulation) observed by RNA-seq across all loci, with expected differences in magnitude owing to platform-specific dynamic ranges and normalization (see Fig. 7 for qRT-PCR and supplementary log2FC Table 6, available as supplementary data at *Bioinformatics Advances* online, for RNA-seq).

The concordant changes in expression observed by qRT-PCR and RNA-seq provide a basis for further investigation of the proposed Sau41–RNAIII–*hla* regulatory relationship. One hypothesis is that Sau41 acts as an RNA sponge, modulating RNAIII availability to activate *hla* translation under biofilm conditions. Testing this hypothesis will require directly assessing Sau41–RNAIII binding and its effects on *hla* translation and α-hemolysin production.

Changes in *walK* align with envelope-remodeling demands typical of sessile growth, while *purR* shifts are compatible with the nucleotide-metabolism reprogramming. Adjustments in *sdhB* and *adhE* hold together with metabolic adaptation to biofilm microenvironments (enhanced fermentative flux and altered TCA activity).

Finally, sRNA4 and Teg13 exhibited concordant qRT-PCR and RNA-seq directions and, as noted earlier, were upregulated in biofilm across all six strains, supporting their prioritization and inferred roles within the predicted regulatory network (see Fig. 6 and Supplementary log2FC Table 6, available as supplementary data at *Bioinformatics Advances* online).

## 4 Conclusions

We present the first genome-wide annotation of noncoding sRNAs in the *S. aureus* ST239 Bmb9393 strain together with a standardized cross-lineage catalog spanning ST239 and US lineages, providing a resource for comparative analyses of sRNA repertoires and regulatory networks. Differential expression between biofilm and planktonic conditions revealed both conserved and lineage-specific transcriptional programs, with 10 sRNAs consistently upregulated and four consistently downregulated across all six strains. Coexpression analysis identified 23 modules, 7 of which were significantly associated with biofilm. Within these modules, Sau41 and RNAIII were coassigned, providing a basis for further investigation of a strain- and context-dependent regulatory model in Bmb9393.

Our integrative prioritization framework combined sequence-based target prediction, differential expression, and coexpression network information to progressively reduce >1.7 million predicted interactions to a final set of 1290 prioritized sRNA–mRNA pairs under the calibrated scheme. Stepwise analyses documented how sequence-based thresholds, biological-context filters, and target-level partner selection contributed to candidate reduction. Comparisons across alternative threshold schemes showed nested target retention, as expected from the workflow design, but substantial sensitivity to thresholds in individual sRNA–mRNA assignments.

The resulting interaction set highlights candidate regulators associated with biofilm formation, cell-envelope homeostasis, virulence, metabolic adaptation, and stress responses, providing hypotheses for future functional studies. The open-source graphical application supports integration, filtering, and prioritization from documented processed inputs and exports intermediate results to facilitate traceability and reuse. Its configurable modules allow application to additional strains, experimental conditions, and other bacterial species.

## Supplementary Material

vbag309_Supplementary_Data

## Data Availability

The source code for the graphical application, analysis scripts, supporting documentation, and a reduced example dataset are publicly available in the project’s GitHub repository: https://github.com/carolina5massena/Integrative-RNA-Seq-and-Co-expression-Analyses-Uncover-Confident-sRNA. The repository also provides access to the complete processed input dataset, comprising the prediction table, six differential-expression tables, and WGCNA node and edge files, together with reference copies of the intermediate output tables generated at each analysis step. The raw RNA-seq data have been deposited in the NCBI sequence read archive (SRA) under BioProject accession PRJNA1327938 and BioSample accessions SAMN51298314–SAMN51298321.
