# Supplementary material for: Transcriptional profiling and coexpression integration for the filtering of relevant bacterial sRNA–mRNA interactions: application to Staphylococcus aureus biofilm
Source: Bioinform Adv. 2026 Oct 13;6(1):vbag309. doi: 10.1093/bioadv/vbag309 (PMC13768106; doi:10.1093/bioadv/vbag309)
Supplement: Supplementary file 1 [file vbag309_supplementary_data.zip › Supplementary_captions.pdf]

Supplementary material

Ribeiro et al.

Transcriptional Profiling and Co-expression Integration for the Filtering of Relevant Bacterial sRNA–mRNA Interactions: Application to *Staphylococcus aureus* Biofilm

Supplementary table descriptions

Tables S1–S20 are supplied as individual editable CSV files. Tables S18–S20 are also reproduced below for convenient reading.

Supplementary Tables S1–S5. Categorization of sRNA genomic localization across strains. Each table presents the classification of sRNAs according to their genomic annotation (antisense, intragenic, intergenic, 5' UTR, and 3' UTR). Tables S1–S5 correspond to Bmb9393, MRSA252, MW2, LAC, and N315, respectively. One table is provided per strain, except for USA500, whose annotation was propagated from USA300, as reported in the original study (Tomlinson, Malof and Shaw, 2021).

Supplementary Tables S6–S11. Differential gene expression (DEG) between biofilm and planktonic conditions. Tables S6–S11 correspond to Bmb9393, MW2, LAC, MRSA252, N315, and NRS385, respectively. Tables list log2 fold changes (log2FC) for each strain, with positive values indicating higher expression in biofilm relative to planktonic growth.

Supplementary Table S12. Cross-lineage module assignment of genes from co-expression analysis. For each row, the table lists the locus IDs for Bmb9393 (ST239), N315 (USA100), MRSA252 (USA200), LAC (USA300/USA500), and MW2 (USA400); the final column reports the WGCNA module (color name) assigned to that locus. The combined gene identifier is preserved alongside strain-specific locus columns and module assignment. For CDS rows, blank strain columns indicate that no corresponding locus tag was provided. sRNA-only identifiers remain in the combined identifier column; blank strain columns in these rows do not indicate absence.

Supplementary Table S13. Hub genes identified within co-expression modules. For each strain, hub genes are listed along with their unique IDs, module membership, correlations of modules with biofilm/planktonic states, and, when available, gene product, protein ID, GO ID, and associated GO terms.

Supplementary Table S14. Gene Ontology (GO) enrichment of co-expression modules. GO terms associated with individual modules, highlighting biological functions enriched among the genes within each module.

Supplementary Table S15. High-priority candidate interactions under calibrated threshold scheme. List of candidate regulatory pairs retained after all filtering steps (see Methods). Because of these filters—including cross-tool thresholds, WGCNA co-assignment, and cross-strain expression-consistency—not all antisense (cis) sRNA–CDS pairs were retained; antisense pairs that failed any criterion were excluded and therefore do not appear here.

Supplementary Table S16. List of candidate interactions under restrictive threshold scheme.

Supplementary Table S17. List of candidate interactions under highly restrictive threshold scheme.

Supplementary Table S18

Metadata of transcriptome samples used in this study. This table lists the RNA-seq samples from GEO Series GSE163153, including their unique Sample ID (GSM accession) and the corresponding S. aureus strains (e.g., N315, MRSA252, USA300$_{L}AC, MW2, NRS385$).

Sample ID	Strain	Treatment	Time	Source
SAMN51298314	Bmb9393	Biofilm	24 h	This work
SAMN51298315	Bmb9393	Biofilm	24 h	This work
SAMN51298316	Bmb9393	Biofilm	24 h	This work
SAMN51298317	Bmb9393	Biofilm	24 h	This work
SAMN51298318	Bmb9393	Planktonic	24 h	This work
SAMN51298319	Bmb9393	Planktonic	24 h	This work
SAMN51298320	Bmb9393	Planktonic	24 h	This work
SAMN51298321	Bmb9393	Planktonic	24 h	This work
GSM4973293	N315	Biofilm	24 h	10.1099/mgen.0.000598
GSM4973294	MRSA252	Biofilm	24 h	10.1099/mgen.0.000598
GSM4973295	USA300_LAC	Biofilm	24 h	10.1099/mgen.0.000598
GSM4973296	MW2	Biofilm	24 h	10.1099/mgen.0.000598
GSM4973297	NRS385	Biofilm	24 h	10.1099/mgen.0.000598
GSM4973298	N315	Planktonic	24 h	10.1099/mgen.0.000598
GSM4973299	MRSA252	Planktonic	24 h	10.1099/mgen.0.000598
GSM4973300	USA300_LAC	Planktonic	24 h	10.1099/mgen.0.000598
GSM4973301	MW2	Planktonic	24 h	10.1099/mgen.0.000598
GSM4973302	NRS385	Planktonic	24 h	10.1099/mgen.0.000598

Supplementary Table S19

List of real-time quantitative reverse transcription polymerase chain reaction primers.

Primer	Direction	Sequence (5'–3')	Reference
sRNA4	Forward	CTGGTCGTGATGGTATGGAAGT	This study
sRNA4	Reverse	GTCTTGTGCTGGTTGTGAGTA	This study
RNAIII	Forward	TGGTTATTAAGTTGGGATGGCT	This study
RNAIII	Reverse	GGAAGGAGTGATTTCAATGGC	This study
walK	Forward	GCGTCGTGAATTTGTTGCCA	This study
walK	Reverse	CGTTCTGTTTCTTCACGGGT	This study
Sau41	Forward	AAAGCAAAGCCACCATCCCT	This study
Sau41	Reverse	AGGAGGACAAAACGATGGCT	This study
purR	Forward	AGACAACAAGGTGACTGAAGGT	This study
purR	Reverse	GAGCCACCAGCCCTCATAAA	This study
adhE	Forward	AGCGGTGTAGGTTCAGACTA	This study
adhE	Reverse	TGTATGGATAACGGCTGTGTGT	This study
sdhB	Forward	ACCACGTATGCCAGAGAAAA	This study
sdhB	Reverse	TGGGTGCAAATTAAACAAACGA	This study
hla	Forward	TGATAGAGATTCTTGGAACCCG	This study
hla	Reverse	AACTGTAGCGAAGTCTGGTGA	This study
Teg13	Forward	CAGACTTGGGGTTATTTATGTGTGT	This study
Teg13	Reverse	ATGTGGTGGCAAGAAGGGAT	This study
rrs	Forward	AGAGATAGAGCCTTCCCCTT	Ferreira et al. (2012)
rrs	Reverse	TTAACCCAACATCTCACGACA	Ferreira et al. (2012)

sRNA names follow the most common name in the Staphylococcal Regulatory RNAs Database (`https://srd.genouest.org`).

Supplementary Table S20

Predicting Interaction Values for sRNA-mRNA Pairs Reported in the Literature. P-values from TargetRNA3, INTARna, RNAplex, and Probabilities from sRNARFtarget.

sRNA	Gene	sRNAFT Target	Target RNA3	Inta RNA	RNAplex	Evidence and DOI
artR	sarT	0.48	0.21	0.1	0.6	Several approaches with artR mutant and wild type. DOI: 10.1007/s00430-013-0307-0
rsaA	mgrA	0.6	0.16	0.35	0.47	Several approaches with rsaA mutant and wild type. DOI: 10.1093/nar/gkx219
rsaA	ssaA	0.58	0.02	0.07	0.47	Several approaches with rsaA mutant and wild type. DOI: 10.1093/nar/gkx219
rsaA	flr	0.6	0.03	0.06	0.29	Several approaches with rsaA mutant and wild type. DOI: 10.1093/nar/gkx219
rsaD	alsS	0.53	0.13	0.04	0.4	RsaD integrates nutritional and environmental stress signals. Experiment during weak acid stress. DOI: 10.1111/mmi.14418
rsaE	rocF	0.57	0.42	0.08	0.06	S.aureus strains were grown aerobically with several antibiotics. DOI: 10.1093/nar/gky584
rsaE	opp3a/3b	0.58	0.15	0.06	0.02	S.aureus strains were grown aerobically with several antibiotics. DOI: 10.1093/nar/gkq462
rsaE	rocD	0.55	0.37	0.1	0.01	S.aureus strains were grown aerobically with several antibiotics. DOI: 10.1093/nar/gky584
rsaE	sucD	0.55	0.04	0.14	0.04	S.aureus strains were grown aerobically with several antibiotics. DOI: 10.1093/nar/gkp668
rsaE	sucC	0.57	0.04	0.12	0.02	S.aureus strains were grown aerobically with several antibiotics. DOI: 10.1093/nar/gkp668
rsaE	sa0873	0.59	0.07	0.05	0.03	S.aureus strains were grown aerobically with several antibiotics. DOI: 10.1093/nar/gky584
rsaE	icaR	0.58	0.37	0.08	0.07	DOI: 10.1093/nar/gky584
rsaI	icaR	0.46	0.4	0.04	0.71	Expression upon glucose limitation in *S. aureus*. DOI: 10.15252/embj.201899363
RNAIII	coa	0.57	0.18	0.41	0.66	During growth phases and a combination of approaches in vivo and in vitro. DOI: 10.1371/journal.ppat.1000809
RNAIII	ecb	0.48	0.25	0.15	0.68	DOI: 10.1042/BC20070137
RNAIII	eap/map	0.45	0.19	0.1	0.54	Mutant and WT and in vitro assays. DOI: 10.1016/j.febslet.2011.02.021
RNAIII	esxA	0.48	0.7	0.13	0.65	sRNA-RNA interaction under conditions that mimic the host environment. DOI: 10.1038/s41467-022-31173-y
RNAIII	hla	0.52	0.5	0.31	0.53	sRNA-RNA interaction in growth phase. DOI: 10.1002/j.1460-2075.1995.tb00136.x
RNAIII	ltaS	0.53	0.09	0.19	0.62	Mutant and WT and in vitro assays in growth phase. DOI: 10.1002/jobm.201400313
RNAIII	murQ	0.53	0.58	0.36	0.56	Mutant and WT in vitro growth with vancomycin. DOI: 10.1038/s41467-022-31177-8
RNAIII	rot	0.49	0.03	0.1	0.67	Mutant and WT in vitro growth without stress. DOI: 10.1111/j.1365-2958.2006.05292.x
RNAIII	sbi	0.51	0.67	0.1	0.64	Mutant and WT in vitro growth without stress. DOI: 10.1093/nar/gku119
RNAIII	spa	0.5	0.2	0.24	0.5	Mutant and WT, in vitro and in vivo growth without stress. DOI: 10.1038/sj.emboj.7600572

sRNA	Gene	sRNAFT Target	Target RNA3	Inta RNA	RNAplex	Evidence and DOI
RNAIII	lytM	0.56	0.17	0.24	0.6	Mutant and WT in vitro growth without stress. DOI: 10.1002/jobm.201100426
sprD	sbi	0.5	0.4	0.18	0.76	During growth phases and in an in vivo model. DOI: 10.1371/journal.ppat.1000927
SprX1	clfB	0.5	0.04	0.16	0.27	Overproduction, mutant and WT, gel mobility shift assay, and in vivo model infection. Early stage of biofilm formation and SprX1 overexpression in Newman strain. DOI: 10.1007/s00430-016-0467-9
SprX2	ecb	0.46	0.14	0.12	0.43	In vivo reporter assay for sRNA- directed gene control. DOI: 10.1093/nar/gkx190
SprX	spoVG	0.52	0.89	0.23	0.53	Regulatory RNAs in the response to biocides, mutant and WT. DOI: 10.1038/s41598-021-86376-y
SprX	walR	0.51	0.1	0	0.56	SprX knockdown strain and mobility shift assay. DOI: 10.1016/j.micres.2021.126785
Teg41	psmα	0.52	0.44	0.28	0.42	Overproduction, mutant and WT of Teg41, and in an abscess model of infection. DOI: 10.1128/mBio.02484-18
Teg49	sarA	0.55	0	0.13	0.49	RNA-seq, RNA, northern blot hybridization, and RNA stability assay of Teg49 mutant and WT. DOI: 10.1111/mmi.14919

Supplementary Figure S1

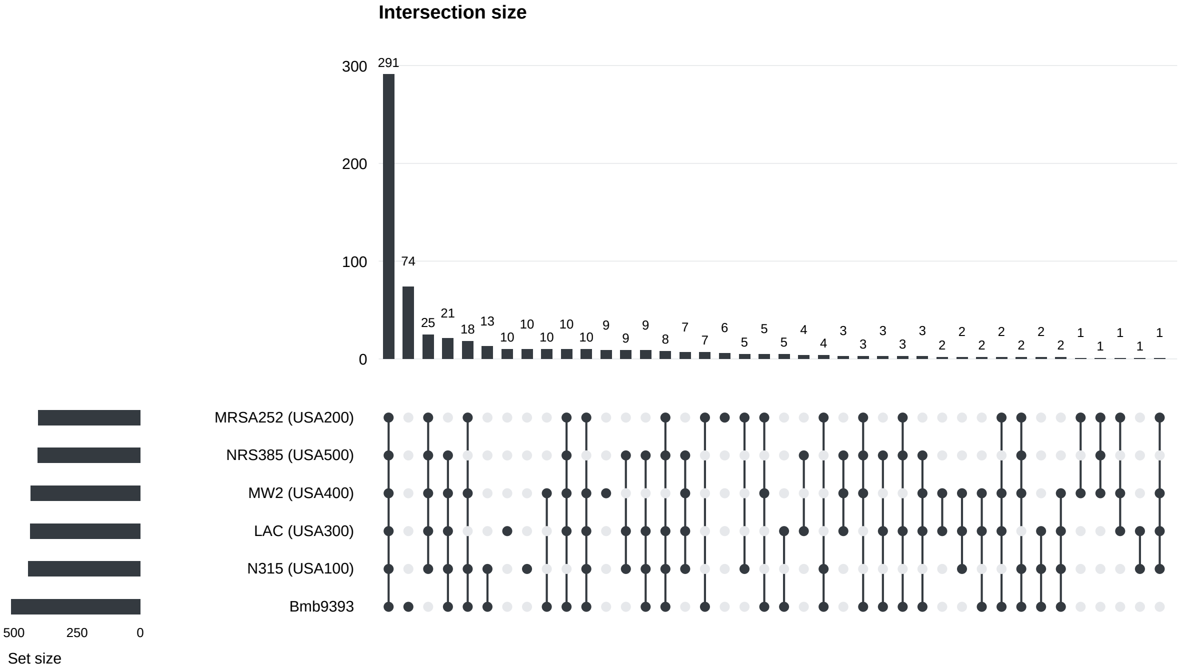

Quantification of the sRNAs with normalized expression levels. (TMM-normalized, converted to RPKM) exceeding 10 reads across the studied strains. The horizontal bars on the left represent the total number of sRNAs detected in each strain, while the vertical bars on the top denote the number of sRNAs shared between specific combinations of strains. The connected dots in the matrix below indicate the strain combinations contributing to each intersection set.

Supplementary Figure S2

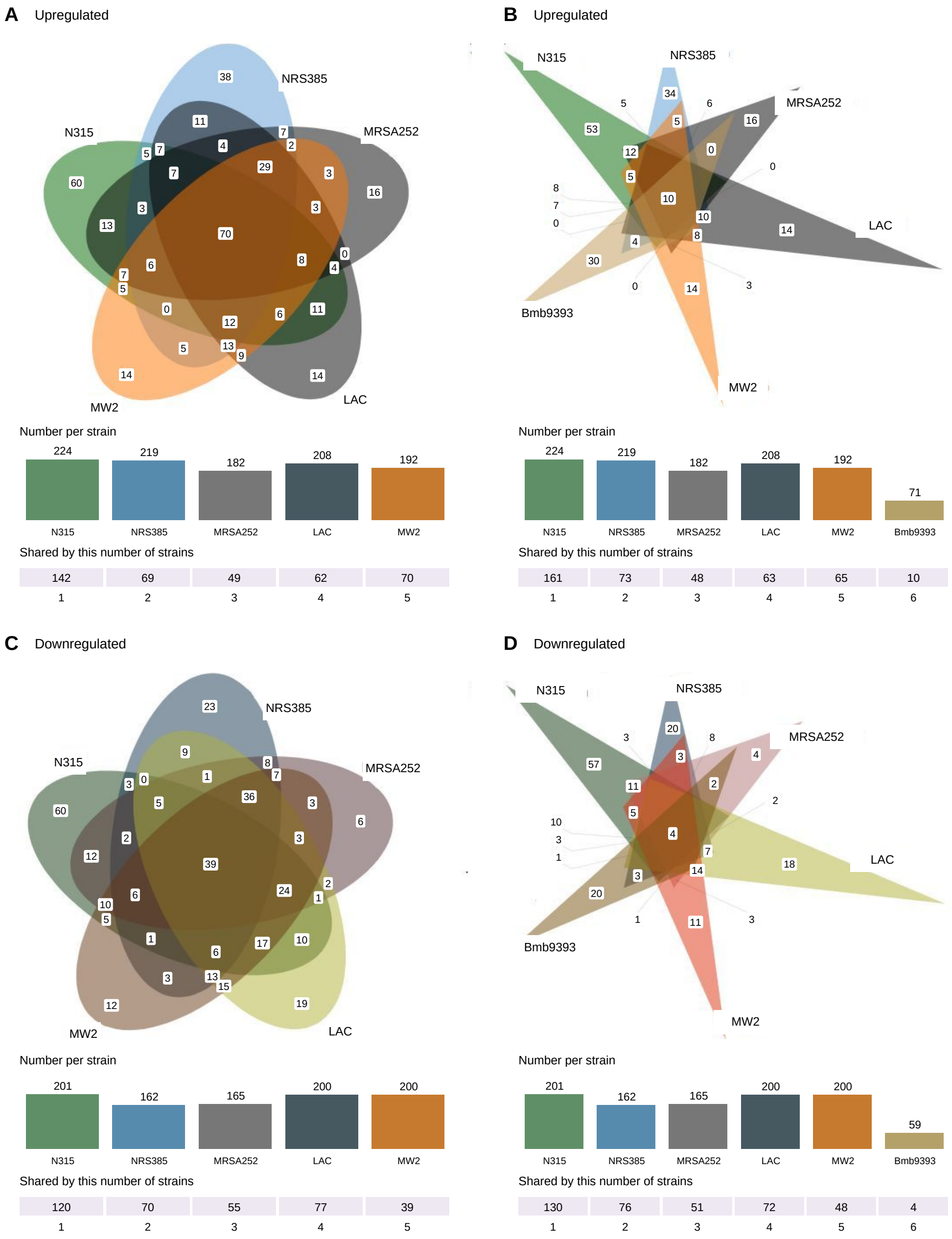

Differentially expressed sRNAs across strains. (A, B) sRNAs upregulated in biofilm relative to planktonic conditions; (C, D) downregulated sRNAs. (A, C) Five USA-lineage strains (N315, NRS385, MRSA252, LAC, and MW2); (B, D) all six strains, including Bmb9393. Bar charts below each Venn diagram show the number of differentially expressed sRNAs per strain. Purple cells report the numbers unique to one strain or shared across increasing numbers of strains, from left to right. Ten upregulated and four downregulated sRNAs are shared by all six strains.
